# The HLTF–PARP1 interaction in the progression and stability of damaged replication forks caused by methyl methanesulfonate

**DOI:** 10.1038/s41389-020-00289-5

**Published:** 2020-12-07

**Authors:** Jia-Lin Shiu, Cheng-Kuei Wu, Song-Bin Chang, Yan-Jhih Sun, Yen-Ju Chen, Chien-Chen Lai, Wen-Tai Chiu, Wen-Tsan Chang, Kyungjae Myung, Wen-Pin Su, Hungjiun Liaw

**Affiliations:** 1 Department of Life Sciences, National Cheng Kung University, No.1 University Road, Tainan City, 701 Taiwan; 2 Institute of Clinical Medicine, College of Medicine, National Cheng Kung University, No.138, Sheng Li Road, Tainan City, 704 Taiwan; 3 Institute of Molecular Biology, College of Life Science, National Chung Hsing University, No.145 Xingda Rd. South Dist., Taichung City, Taiwan; 4 Department of Biomedical Engineering, National Cheng Kung University, Tainan City, Taiwan; 5 Department of Biochemistry and Molecular Biology, College of Medicine, National Cheng Kung University, Tainan City, Taiwan; 6 IBS Center for Genomic Integrity, UNIST-gil 50, Ulsan, 689-798 Republic of Korea; 7 Departments of Oncology and Internal Medicine, National Cheng Kung University Hospital, College of Medicine, National Cheng Kung University, Tainan City, 704 Taiwan

**Keywords:** DNA damage and repair, DNA replication, Genomic instability

## Abstract

Human HLTF participates in the lesion-bypass mechanism through the fork reversal structure, known as template switching of post-replication repair. However, the mechanism by which HLTF promotes the replication progression and fork stability of damaged forks remains unclear. Here, we identify a novel protein–protein interaction between HLTF and PARP1. The depletion of HLTF and PARP1 increases chromosome breaks, further reduces the length of replication tracks, and concomitantly increases the number of stalled forks after methyl methanesulfonate treatment according to a DNA fiber analysis. The progression of replication also depends on BARD1 in the presence of MMS treatment. By combining 5-ethynyl-2′-deoxyuridine with a proximity ligation assay, we revealed that the HLTF, PARP1, and BRCA1/BARD1/RAD51 proteins were initially recruited to damaged forks. However, prolonged stalling of damaged forks results in fork collapse. HLTF and PCNA dissociate from the collapsed forks, with increased accumulation of PARP1 and BRCA1/BARD1/RAD51 at the collapsed forks. Our results reveal that HLTF together with PARP1 and BARD1 participates in the stabilization of damaged forks, and the PARP1–BARD1 interaction is further involved in the repair of collapse forks.

## Introduction

Despite the fact that cells have evolved several DNA repair mechanisms to repair DNA damage, it is almost inevitable that some DNA lesions will escape these repair mechanisms and collide with active replication forks. As a result, replication forks stall and the prolonged stalling of replication forks can cause them to collapse, leading to DSBs and genomic instability^1,2^.

In prokaryotic cells, stalled or collapsed replication forks are reactivated by recombination-dependent pathways^3^. In eukaryotic cells, stalled forks are reactivated by post-replication repair (PRR)^4^. PRR is highly conserved from yeast to humans and it can be divided into two sub-pathways: translesion synthesis (TLS) and template switching (TS). Although it is still not clear how the choice of pathways is determined in mammalian cells, the monoubiquitination and polyubiquitination of PCNA direct the damaged forks to either the TLS or TS pathway, respectively. The TLS pathway involves PCNA monoubiquitination on lysine-164 by the E2 ubiquitin-conjugating enzyme RAD6 and E3 ubiquitin ligase RAD18. Then the monoubiquitinated PCNA recruits the low-fidelity TLS polymerases to synthesize DNA, bypassing the damaged sites^5–8^. The TS pathway involves the K63-linked polyubiquitination of PCNA mediated by the E2 ubiquitin-conjugating enzyme UBC13 and MMS2, as well as the E3 ubiquitin ligase SHPRH and HLTF (both SHPRH and HLTF are homologs of yeast *RAD5*) onto monoubiquitinated PCNA^9–13^. Importantly, HLTF has been shown to promote strand invasion or fork reversal activities^14–18^. The structure of reversed forks is finally observed by using electron microscopy^19,20^. In addition to HLTF, SMARCAL1 and ZRANB3 are also able to convert stalled forks into reversed forks^21–24^, and the formation of reversed forks depends on RAD51 recombinase^19,20^. However, it remains unclear whether these helicases work together, act alone, or depend on the types of DNA lesions.

PARP1 is an ADP-ribosyl transferase enzyme that catalyzes the formation of poly(ADP-ribose) (PAR) polymers onto its target proteins^25^. Previous studies have revealed that PARP1 can bind to DNA strand breaks and participate in base/nucleotide excision repair and DSB repair^26–31^. The activation of PARP1 at break sites leads to auto-modification and the modification of other target proteins with PAR polymers^32^. Additionally, PARP1 is also activated by stalled forks^27^, however, the underlying mechanism remains unclear.

Nevertheless, little is known about the TS pathway, such as how it is induced and executed. In this study, we identify a novel protein–protein interaction between HLTF and PARP1. HLTF and PARP1 assist in replication progression and stability of the damaged forks, possibly by forming reversed forks, and this process also depends on homologous recombination (HR). By using an EdU-PLA assay, we provide a valuable kinetic study to reveal the loading of PCNA, HLTF, PARP1, and HR proteins onto damaged forks. Our results reveal that HLTF together with PARP1 and HR proteins participate in replication progression and fork stability of damaged forks. When the damaged forks collapse, PARP1 and HR proteins go on to repair the collapsed forks.

## Results

### HLTF interacts with PARP1

**Fig. 1:**
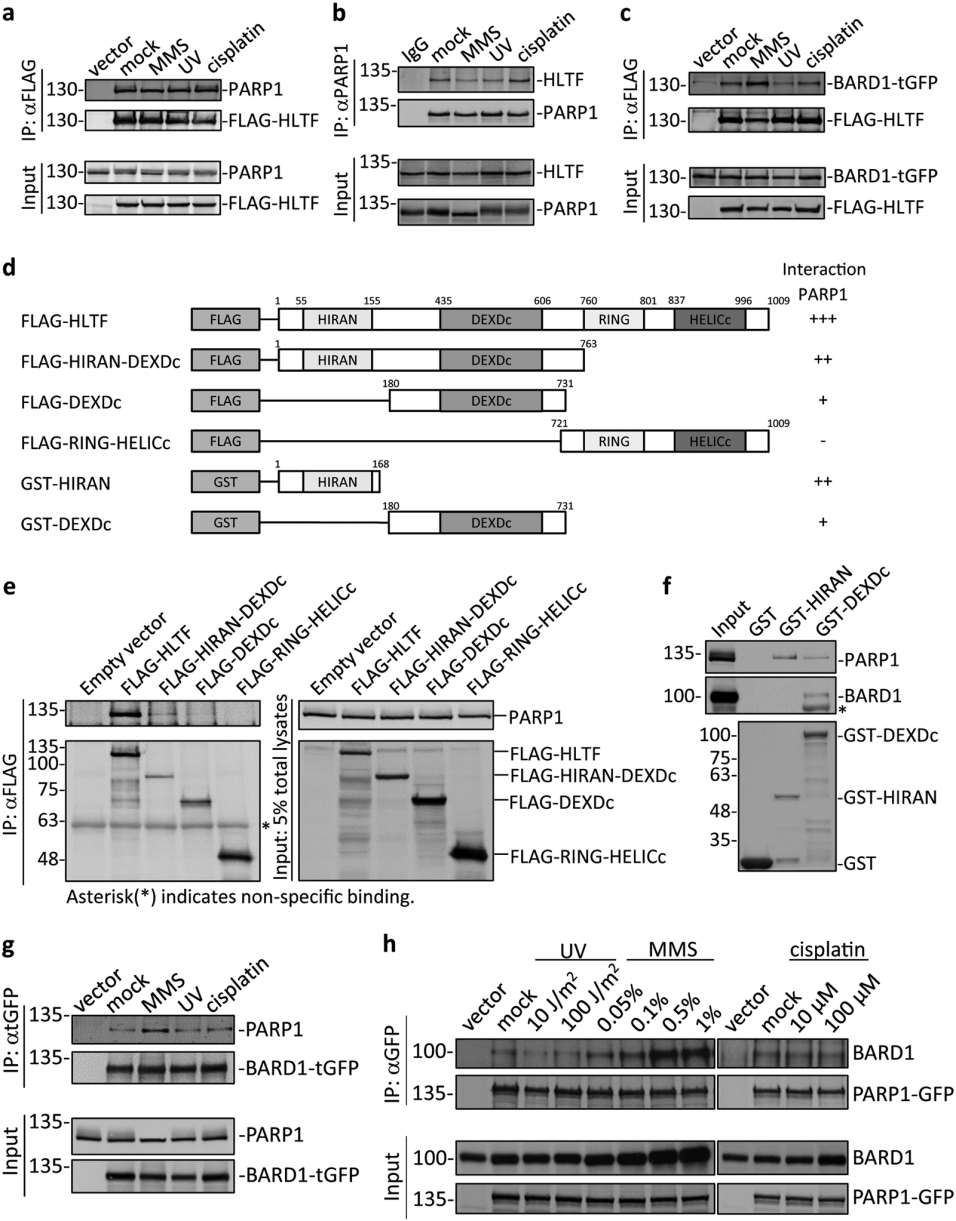
HLTF interacts with PARP1 and BARD1. **a** HLTF interacts with PARP1. HEK293T cells were transfected with plasmids containing the FLAG–HLTF fusion gene and were subjected to 1.2 mM (0.01%) MMS, 60 J/m^2^ UV, and 5 μM cisplatin treatments for 3 h. The same treatment was applied in (**b**, **c**, **g**). The FLAG–HLTF complex was immunoprecipitated with an anti-FLAG antibody, and proteins associated with HLTF were detected by immunoblotting. The empty vector p3xFLAG was used as the negative control. Input represents 5% of total cell lysates. **b** The endogenous protein–protein interaction between PARP1 and HLTF. The endogenous PARP1 was immunoprecipitated with an anti-PARP1 antibody and the pull-down HLTF was detected by anti-HLTF antibodies. Input represents 5% of total cell lysates. **c** HLTF interacts with BARD1. HEK293T cells were transfected with plasmids containing the FLAG–HLTF and BARD1–turboGFP fusion genes. The FLAG–HLTF complex was immunoprecipitated with an anti-FLAG antibody, and proteins associated with HLTF were detected by immunoblotting. The empty vector p3xFLAG was used as the negative control. Input represents 5% of total cell lysates. **d** The schematic representation of HLTF constructs. **e** The domains of HLTF that interact with PARP1. HEK293T cells were transfected with various constructs of FLAG–HLTF. The cell lysates were immunoprecipitated with an anti-FLAG antibody, and the immunoprecipitates were then subjected to immunoblotting. Input represents 5% of total cell lysates. Asterisk is nonspecific binding. **f** The GST pull-down assay. The GST fusion proteins were purified from *E. coli Rosetta* (lower panel) and were then incubated with cell lysates derived from HEK293T cells. The proteins associated with the GST fusion proteins were detected by antibodies as indicated (upper panel). The GST proteins were used as a control. Input represents 5% of total cell lysates. The asterisk indicates nonspecific binding. **g** BARD1 interacts with PARP1. HEK293T cells were transfected with plasmids containing the BARD1–turboGFP fusion gene. The BARD1–turboGFP complex was immunoprecipitated with an anti-turboGFP (tGFP) antibody. Input represents 5% of total cell lysates. **h** PARP1 interacts with BARD1. HEK293T cells were transfected with plasmids containing the PARP1–GFP fusion gene and were treated with UV, MMS, and cisplatin for 3 h. The PARP1–GFP complex was immunoprecipitated with an anti-GFP antibody, and proteins associated with PARP1 were detected by immunoblotting. The empty vector pEGFP-N1 was used as the negative control. Input represents 5% of total cell lysates.

Several lines of evidence have suggested that HLTF promotes a fork reversal structure by pairing nascent DNA with its sister chromatid in the TS pathway^14–18^. In order to identify other proteins that are involved in the TS pathway, we performed a proteomic analysis of the HLTF complex. We purified the HLTF complex from FLAG–HLTF-expressing HEK293 cells. The associated proteins were subjected to mass spectrometry. We identified PARP1 as one of interacting factors (Supplementary Table S1). To verify the interactions, we performed a coimmunoprecipitation assay. We transfected HEK293T cells with a plasmid containing FLAG-tagged HLTF (FLAG–HLTF). FLAG–HLTF was then immunoprecipitated with an anti-FLAG antibody. As shown in Fig. 1a, HLTF interacted with PARP1. We also treated cells with various DNA damaging agents, including MMS, UV, and cisplatin. None of these DNA damaging treatments enhanced the interaction (Fig. 1a).

To determine whether the interaction between HLTF and PARP1 exists at the endogenous level, we immunoprecipitated endogenous PARP1 with a specific anti-PARP1 antibody. PARP1 was able to pull down endogenous HLTF (Fig. 1b). Similarly, exposure to DNA damaging agents did not enhance the interaction (Fig. 1b). Additionally, we also performed a proximity ligation assay (PLA) to verify the interaction. PLA measures the association between the two proteins in vivo (Supplementary Fig. S1a). HLTF/PARP1 PLA foci indeed formed in the untreated cells (0 h) (Supplementary Fig. S1b–d). MMS treatment slightly increased the levels of PLA foci at 1-h timepoint, but slightly decreased at 3-h timepoint. Our PLA results suggest that HLTF interacts with PARP1 in the untreated and MMS-treated cells over time.

We next mapped the domains of HLTF that interact with PARP1. We transfected HEK293T cells with various constructs of HLTF containing either the HIRAN-DEXDc, DEXDc, or RING-HELICc domains of HLTF (Fig. 1d). These FLAG–HLTF constructs were immunoprecipitated with an anti-FLAG antibody. The HIRAN-DEXDc domain of HLTF was able to interact with PARP1 (Fig. 1e). This result was also confirmed by a GST pull-down assay. The GST-HIRAN or DEXDc domains of HLTF were purified from *Escherichia coli Rosetta*. The purified GST-HIRAN and DEXDc domains of HLTF can pull down PARP1, with the HIRAN domain having a stronger interaction with PARP1 (Fig. 1f). To the best of our knowledge, this is the first study showing that HLTF interacts with PARP1.

### Both HLTF and PARP1 interact with BARD1

Recent studies have shown that PARP1 interacts with BARD1 through the BRCT domain of BARD1 and the PAR moiety of PARP1 and that this interaction is important for the recruitment of BARD1 to DSB sites^33,34^. To test whether the PARP1–BARD1 interaction also exists at damaged replication forks, we treated the cells with various types of DNA damaging agents, including MMS, UV, and cisplatin. HEK293T cells were transfected with a plasmid containing BARD1 tagged with a turboGFP (BARD1–tGFP) followed by treatment of the cells with these DNA damaging agents. We then performed the immunoprecipitation assay with an anti-tGFP antibody. BARD1 interacted with PARP1 and the interaction was enhanced by MMS treatment, but not by UV or cisplatin treatments (Fig. 1g). Reversely, we also showed PARP1–GFP was able to pull down BARD1 and MMS enhanced the interaction, with higher doses of MMS exhibiting a stronger interaction (Fig. 1h). MMS is an alkylating agent that causes DNA lesions by modifying guanine and adenine to form 7-methylguanine and 3-methyladenine, respectively^35^. Since MMS causes DSBs to occur specifically during S-phase^36^, it suggests that the enhanced interaction between PARP1 and BARD1 occurs at damaged replication forks.

Since HLTF interacts with PARP1, we also tested whether HLTF interacts with BARD1. By using coimmunoprecipitation experiment, FLAG–HLTF was able to immunoprecipitate BARD1–tGFP (Fig. 1c). We further tested whether the HLTF–BARD1 interaction exists in vivo. HLTF/BARD1 PLA foci indeed were shown in nucleus in the untreated cells and MMS treatment significantly increased the levels of HLTF/BARD1 PLA foci at 3-h timepoint (Supplementary Fig. S1b–d). Additionally, we also found that the levels of PARP1/BARD1 PLA foci were significantly increased at 3-h timepoint following MMS treatment (Supplementary Fig. S1b–d). We noticed that the interaction of BARD1 either with HLTF or PARP1 showed lower levels of PLA foci, indicating that the interaction of BARD1 with HLTF and PARP1 could be transient.

We further mapped the domains of HLTF that interact with BARD1. The purified GST-DEXDc domain, but not the HIRAN domain of HLTF, can pull down BARD1 (Fig. 1f). Therefore, we conclude that HLTF is able to interact with PARP1 and BARD1.

### HLTF and UBC13 are associated with active replisomes

**Fig. 2:**
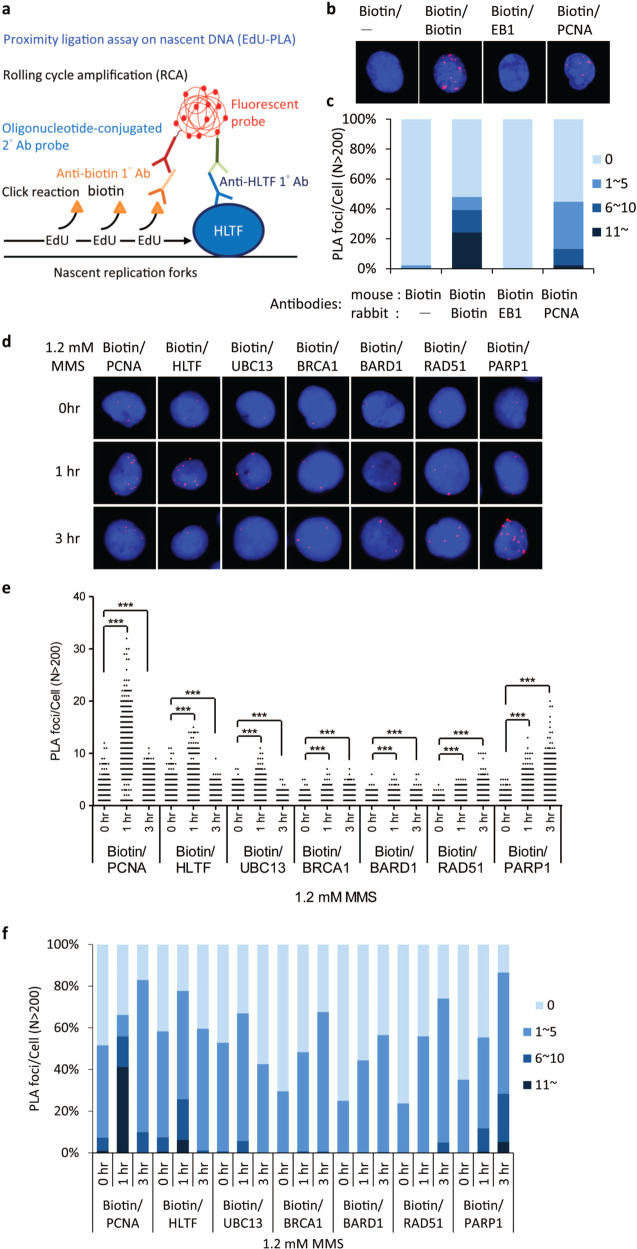
HLTF, PARP1, BARD1, UBC13, and PCNA accumulate at damaged forks. **a** The schematic representation of the EdU-PLA assay. DNA was pulse labeled with EdU to label the nascent DNA. **b** The control experiments for the EdU-PLA assay. Specific antibodies derived from mouse or rabbit are indicated. PLA foci were shown in red; DAPI staining was shown in blue. **c** The quantitative data derived from (**b**). The number of PLA foci from each cell was classified into four groups: 0, 1–5, 6–10, and 11~ foci. At least 200 cells from each condition were measured in the EdU-PLA assay. **d** The representative images of EdU-PLA foci of PCNA, HLTF, UBC13, BRCA1, BARD1, RAD51, and PARP1. Cells were treated with 1.2 mM (0.01%) MMS, and samples were collected at 0-, 1-, and 3-h timepoints following MMS treatment. **e** Distribution of PLA foci from each cell derived from (**d**). At least 200 cells from each condition were measured. The *p* value was determined by the Mann–Whitney test. ***p < 0.001. **f** The percent stacked column graph derived from (**e**). The number of PLA foci from each cell was classified into four groups: 0, 1–5, 6–10, and 11~ foci. The percentage of each group is indicated in the plot. All experiments have been repeated at least twice, with very similar results.

Since we found that HLTF interacts with PARP1 and BARD1 and that there is an enhanced interaction between PARP1 and BARD1, we wanted to investigate whether these interactions occur at damaged replication forks. We therefore performed a kinetic study of HLTF, PARP1, and BARD1 by utilizing an EdU-PLA assay that measures the association of proteins with nascent synthesized DNA^37,38^. Since we found that MMS enhances the PARP1–BARD1 interaction and MMS is also commonly used to study the TS mechanism of PRR^14,39–42^, we used MMS to induce DNA lesions. For these studies, we labeled the human nasopharyngeal carcinoma cell line, HONE6, with EdU for 10 min and subsequently treated with mock or 1.2 mM (0.01%) MMS to induce DNA damage. Biotin was conjugated to EdU by the click reaction. Specific antibodies against biotin and the target protein were used to detect the association of the target protein with replication tracks. If the target protein was associated with replication forks, it shows PLA foci (Fig. 2a). As the negative control in this assay, a single anti-biotin antibody (biotin/−) or microtubule associated protein EB1 (EB1/biotin) failed to show any PLA foci (Fig. 2b, c). In contrast, two anti-biotin antibodies derived from mouse and rabbit (biotin/biotin) showed many PLA foci (Fig. 2b, c). Since PCNA is a cofactor of DNA polymerases that are associated with active replisomes^43^, the anti-PCNA and anti-biotin antibodies (PCNA/biotin) showed many PLA foci (Fig. 2b, c). Indeed, the pattern and the number of PCNA PLA foci were similar to previously published results^38,44^. Therefore, these control experiments validate our EdU-PLA assay.

To determine the kinetics of HLTF at stalled forks after MMS treatment, we monitored the PLA foci formation of HLTF in nascent DNA at 0-, 1-, and 3-h timepoints after MMS treatment. Additionally, since UBC13 is also involved in the TS pathway, we also monitored the PLA foci of UBC13. Interestingly, HLTF, UBC13, and PCNA demonstrated a very similar pattern. Approximately 50% of the cells showed PCNA PLA foci at the 0-h timepoint with PLA foci ranging from 1 to 12, indicating that ~50% of the cells were in S-phase in untreated cells (Fig. 2d–f). HLTF/EdU and UBC13/EdU-PLA foci were also shown in the nucleus, with ~55% and 50% of cells showing PLA foci and the foci number ranging from 1 to 11 and 1 to 7 foci, respectively in the untreated cells (Fig. 2d–f). Since the levels of PLA foci are similar among PCNA, HLTF, and UBC13, it suggests that HLTF and UBC13 were associated with the active replication forks and could be components of replication machinery. Consistent with our results, several groups either by using iPOND (isolation of proteins on nascent DNA) or NCC (nascent chromatin capture) approaches^18,45^, they also conclude that HLTF is part of replication machinery. Here, we further identify UBC13 being a part of replisomes.

Interestingly, MMS treatment increased the number of PCNA, HLTF, and UBC13 PLA foci in the cells at the 1-h timepoint following MMS treatment, with ~70%, 80%, and 70% of cells showing PLA foci and the foci number ranging from 1 to 32, 1 to 15, and 1 to 11 foci, respectively (Fig. 2d–f). Our results suggest that more PCNA, HLTF, and UBC13 proteins were accumulated at damaged forks after 1-h of MMS treatment. Surprisingly, the PCNA, HLTF, and UBC13 PLA foci started to decline at the 3-h timepoint after treatment, with ~80%, 60%, and 50% of cells showing PLA foci and the foci number ranging from 1 to 11, 1 to 9, and 1 to 5 foci, respectively (Fig. 2d–f), indicating that the damaged forks begin to collapse and PCNA, HLTF, and UBC13 proteins dissociate from the collapsed forks.

### HR and PARP1 proteins are enriched at collapsed replication forks

Since PARP1 interacts with HLTF and BARD1, we also tested the dynamic processes of PARP1 and BARD1 at replication forks following the treatment of MMS for 1 and 3 h. Additionally, since BRCA1 and BARD1 form a heterodimer and RAD51 is required for the formation of reversed forks^19,20,46^, we also tested the recruitment of BRCA1 and RAD51 to stalled forks. We observed that the PLA foci of PARP1, BRCA1, BARD1, and RAD51 show different patterns from the PLA foci of HLTF, UBC13, and PCNA (Fig. 2d–f). Approximately 30% of the cells show the PARP1, BRCA1, BARD1, and RAD51 PLA foci (Fig. 2f), and the number of PLA foci ranges from 1 to 5, 1 to 5, 1 to 6, and 1 to 4, respectively (Fig. 2d, e). Therefore, the number of PLA foci is much less than cells with the PCNA, HLTF, and UBC13 PLA foci at the 0-h timepoint (Fig. 2d–f). In response to MMS treatment, cells with the PARP1, BRCA1, BARD1, and RAD51 PLA foci and the number of PLA foci in nucleus increased at the 1-h timepoint, and continued to increase to 3-h timepoint (Fig. 2d–f). Since all of proteins tested here (PCNA, HLTF, UBC13, BRCA1, BARD1, RAD51, and PARP1) were recruited at the 1-h timepoint, the results suggest that these proteins may participate in forming reversed forks to protect or repair damaged forks. At the 3-h timepoint, stalled forks begin to collapse, and simultaneously, PARP1, BRCA1, BARD1, and RAD51 accumulate at the collapsed forks, and possibly repair the collapsed forks by the HR repair pathway.

### The kinetics of PARP1 and HLTF at damaged forks alters in the HLTF- and PARP1-knockout cells

**Fig. 3:**
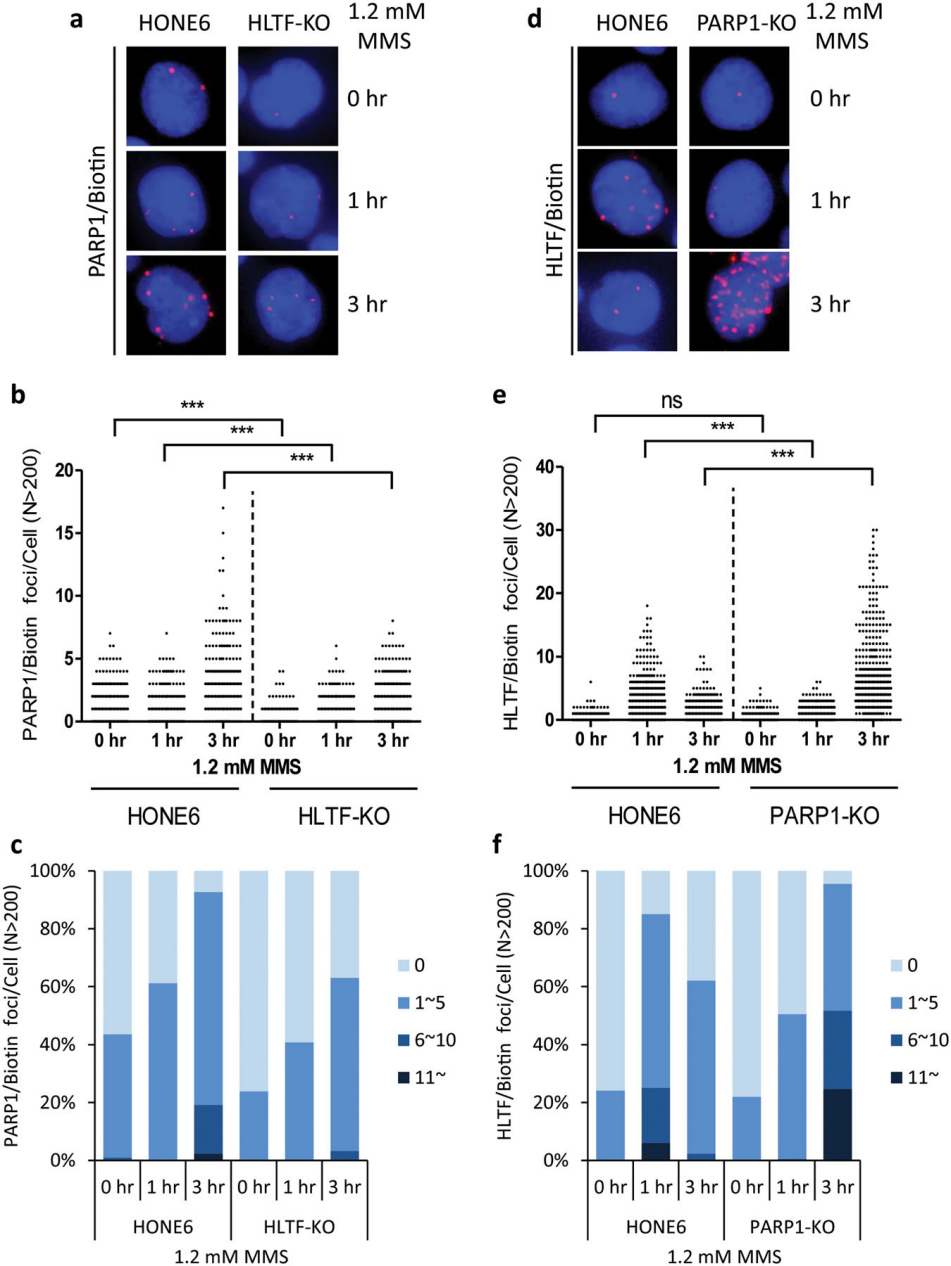
The kinetics of PARP1 and HLTF at damaged forks in the HLTF-KO and PARP1-KO cells. **a** The representative images of PARP1/EdU-PLA foci in wild-type and HLTF-KO HONE6 cells. Cells were treated with 1.2 mM (0.01%) MMS, and samples were collected at 0-, 1-, and 3-h timepoints following MMS treatment. **b** Distribution of PARP1/EdU-PLA foci from each cell derived from (**a**). At least 200 cells from each condition were measured. The *p* value was determined by the Mann–Whitney test. ***p < 0.001. **c** The number of PLA foci from each cell was classified into four groups: 0, 1–5, 6–10, and 11~ foci, and the percentage of each group is indicated in the plot. **d** The representative images of HLTF/EdU-PLA foci. Cells were treated with the same condition as (**a**). **e** Distribution of HLTF/EdU-PLA foci from each cell derived from (**d**). At least 200 cells from each condition were measured. ns no significance; ***p < 0.001 (Mann–Whitney test). **f** The percent stacked column graph derived from (**e**). All experiments have been repeated twice, with very similar results.

To determine the significance of the interaction between HLTF and PARP1, we monitored the abundance of HLTF and PARP1 at replication and damaged forks in PARP1-knockout (PARP1-KO) and HLTF-knockout (HLTF-KO) HONE6 cells. The HLTF and PARP1 genes were deleted by using a CRISPR knockout strategy, and the deletion of HLTF and PARP1 was verified by western blotting (Supplementary Fig. S2). Cells were treated with 1.2 mM (0.01%) MMS, and the recruitment of HLTF or PARP1 was monitored in the untreated cells (0 h), and at 1-h and 3-h timepoint following the treatment. In wild-type HONE6 cells, ~40% of cells showed PARP1/EdU-PLA foci (Fig. 3a, c), and the number of PARP1/EdU-PLA foci ranged from 1 to 7 foci in the untreated cells (0 h) (Fig. 3a, b). MMS treatment increased the number of cells with PLA foci and foci number at 1 h and continued to increase to 3-h timepoint (Fig. 3a–c). In contrast, the levels of PLA foci significantly reduced in the HLTF-KO cells at each timepoint, with 20%, 40%, and 60% of cells showing PLA foci at 0, 1, and 3 h, respectively (Fig. 3c). Since the levels of PARP1 at damaged forks are significantly reduced in the HLTF-KO cells, our results indicate that HLTF could promote the recruitment of PARP1 to damaged forks.

Interestingly, when we monitored the HLTF/EdU-PLA foci in the PARP1-KO cells, the HLTF/EdU-PLA foci showed different kinetics of HLTF from wild-type HONE6 cells (Fig. 3d–f). In wild-type HONE6 cells, the levels of PLA foci increased at 1 h, and then reduced at 3-h timepoint (Fig. 3e, f). In contrast, in the PARP1-KO cells, the levels of PLA foci continued to increase over time following the treatment (Fig. 3d–f). The reduced levels of HLTF/EdU-PLA foci at 1-h timepoint in the PARP1-KO cells also indicate that the HLTF–PARP1 interaction enhances the recruitment of HLTF to damaged forks. However, the high levels of HLTF associated with damaged forks at 3-h timepoint in the PARP1-KO cells indicates that tremendous single-strand breaks (SSBs) occur in damaged forks and HLTF appears to stabilize the broken forks by forming reversed forks.

### Depletion of HLTF, UBC13, BARD1, and PARP1 decreases the length of replication tracks and simultaneously increases the number of stalled forks following MMS treatment

**Fig. 4:**
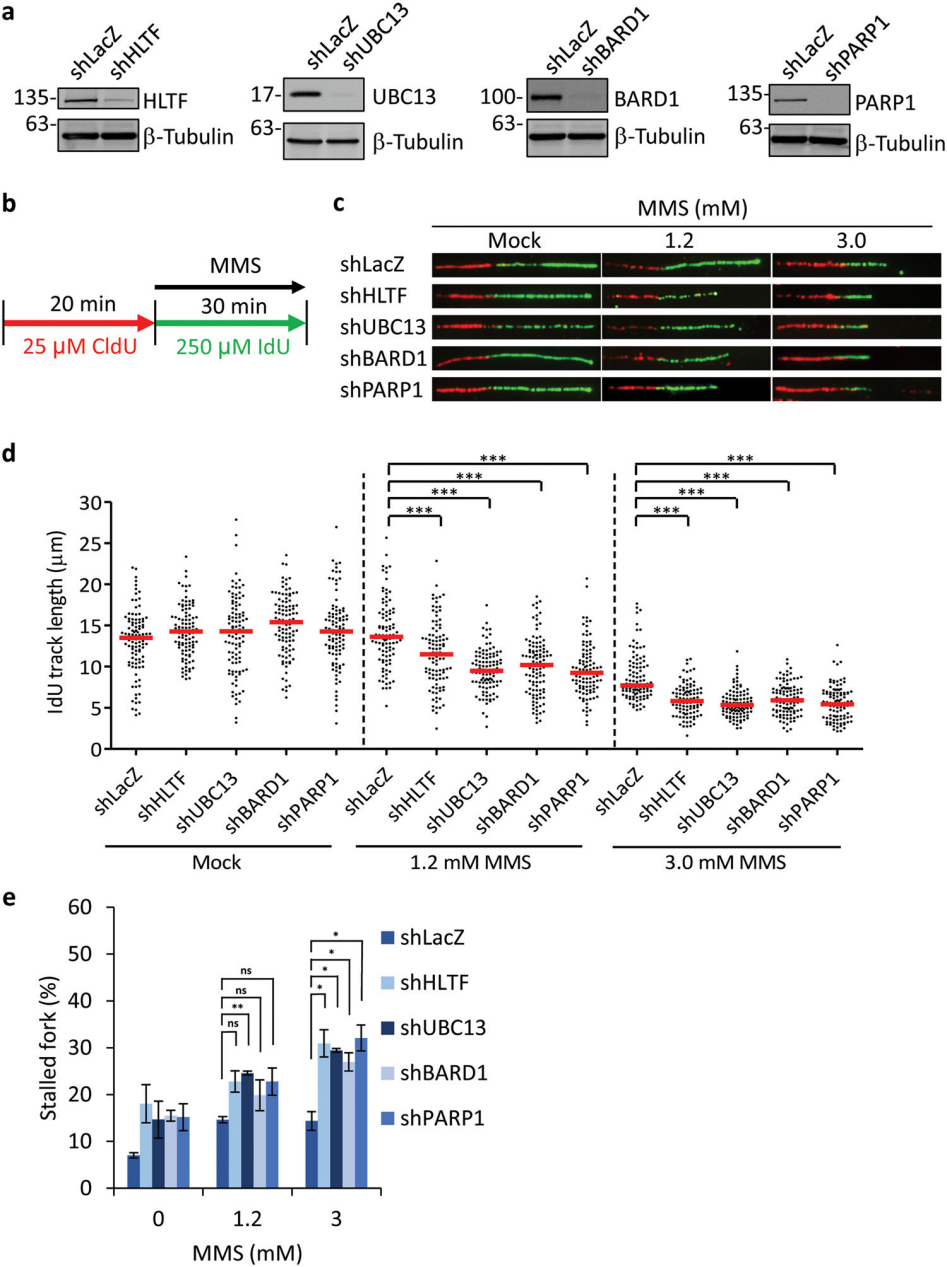
Depletion of HLTF, UBC13, BARD1, or PARP1 significantly decreases replication tracks and increases stalled replication forks in HONE6 cells. **a** The depletion of HLTF, UBC13, BARD1, and PARP1 was verified by western blot analysis with the specific antibodies indicated. **b** Labeling protocols for DNA fiber analysis. **c** Representative images of DNA fibers. **d** Quantitation of IdU track length derived from each cell line treated with various concentrations MMS. ***p < 0.001 (Mann–Whitney test). **e** Quantitation of stalled forks. DNA fiber experiments from each cell line were repeated twice, and it showed similar trends. At least 100 DNA fibers derived from each cell line were measured in these experiments. The *p* value of each test is shown. ns no significance; *p < 0.05; **p < 0.01 (Student’s *t* test).

To determine the function of HLTF, UBC13, BARD1, and PARP1 in replication progression in the presence of MMS-induced lesions, we monitored the progression of DNA replication using a DNA fiber assay. We used shRNA-packed lentivirus to deplete the expression of HLTF, UBC13, BARD1, and PARP1. The non-targeting shRNA, shLacZ, was used as a control. As shown in Fig. 4a, the expression levels of HLTF, UBC13, BARD1, and PARP1 were efficiently depleted by shRNA. Subsequently, these cells were first pulse labeled with 5-chlorodeoxyuridine (CldU) for 20 min, and then treated with both MMS and iododeoxyuridine (IdU) for 30 min (Fig. 4b). DNA spreads were prepared and analyzed by immunofluorescence with specific fluorescent conjugated antibodies against CldU and IdU. We then analyzed the IdU track length distributions after MMS treatment. The IdU track lengths were similar among the wild-type and the HLTF-, UBC13-, BARD1-, and PARP1-depleted cells, suggesting that these proteins are not required for replication elongation per se (Figs. 4c, d and S3). Indeed, MMS treatment reduced the IdU track lengths, and the reduction of track length correlated with the dose of MMS (Fig. 4c, d). These results confirm that DNA lesions are obstacles for the progression of DNA replication. Consistent with these results, MMS treatment increased the number of stalled forks (showing only red tracks) (Fig. 4e). Interestingly, the depletion of HLTF, UBC13, BARD1, and PARP1 exhibited more numbers of stalled forks (Fig. 4e) and further reduced the IdU track lengths after MMS treatment (Fig. 4c, d). Our results suggest that the depletion of HLTF, UBC13, BARD1, or PARP1 further affects the progression of DNA replication in the presence of MMS. Since MMS can cause DSBs specifically at S-phase, the short IdU track length shown in the HLTF-, UBC13-, BARD1-, and PARP1-deficient cells could be due to the fact that DNA replication encounters more SSB templates generated during BER in these gene-deficient cells. As a result, DNA replication terminates due to encountering those broken templates more frequently in the HLTF-, UBC13-, BARD1-, and PARP1-deficient cells. Additionally, since HLTF promotes fork reversal, the broken templates may be secured by the four-way fork reversal structure, thus holding the broken forks from dissociation. It suggests that HLTF, UBC13, BARD1, and PARP1 can stabilize damaged forks and prevent the collapse of forks. Alternatively, we cannot rule out the possibility that HLTF, UBC13, BARD1, and PARP1 are involved in fork restart through the fork reversal structure (see “Discussion”).

### Depletion of HLTF, UBC13, BARD1, and PARP1 increases DSBs in response to MMS-induced DNA lesions

**Fig. 5:**
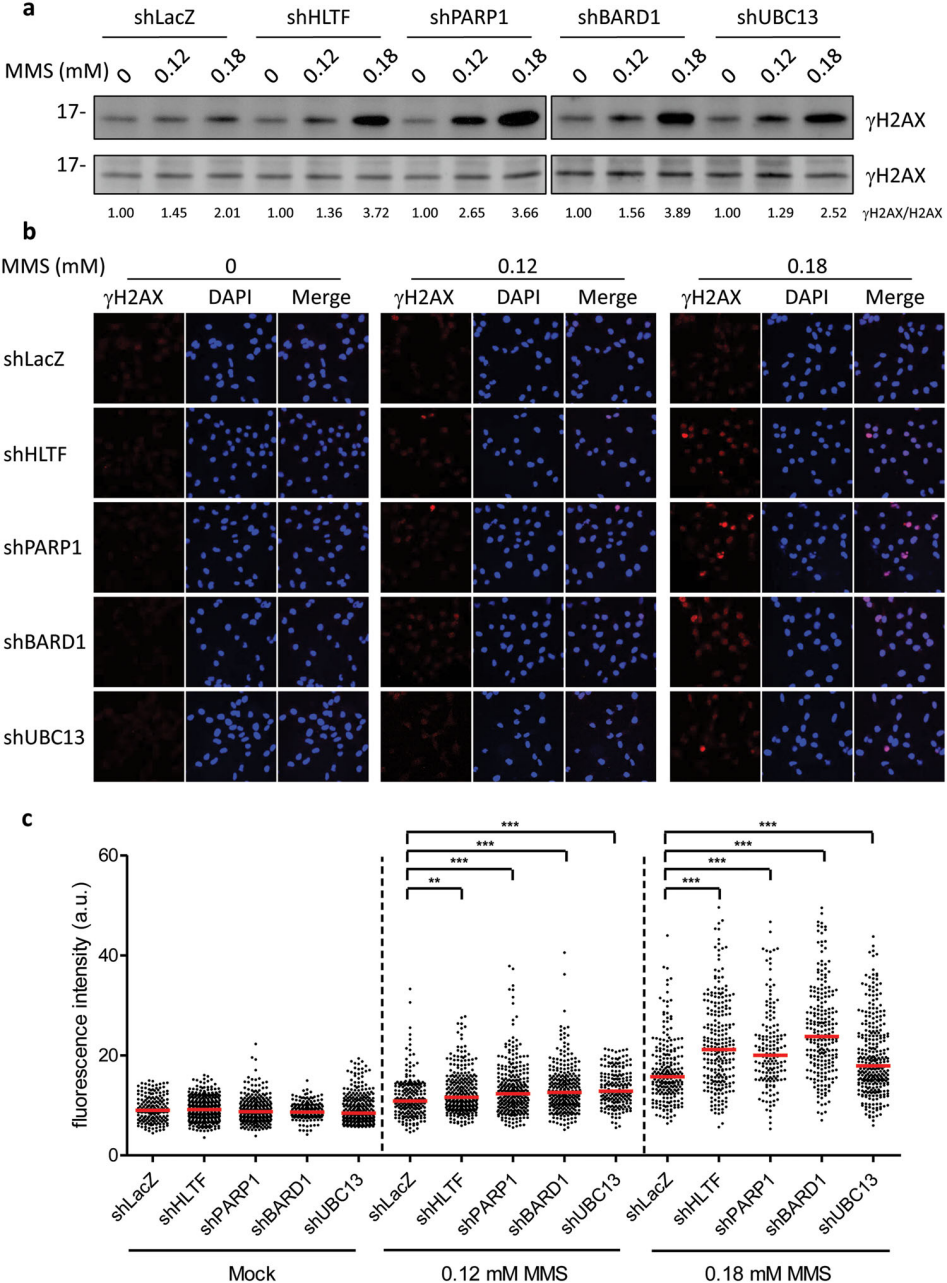
MMS causes more DSBs in HLTF-, PARP1-, BARD1-, and UBC13-depleted HONE6 cells. **a** The western blot of γH2AX in the control cell (LacZ) and HLTF-, PARP1-, BARD1-, and UBC13-depleted cells. Cells were treated with 0.12 or 0.18 mM of MMS for 24 h. Cells were harvested at the indicated timepoints and subjected to western blot analysis with specific antibodies as indicated. **b** The confocal microscopy results of γH2AX derived from each cell line. Cells were chronically treated with 0.12 or 0.18 mM of MMS for 24 h. Cells were fixed and immunostained with γH2AX antibody. **c** The intensity of γH2AX in each cell was quantified using the FV10-ASW software. At least 150 cells from each cell line were quantified. **p < 0.01; ***p < 0.001 (Mann–Whitney test).

To test whether the depletion of HLTF, PARP1, and BARD1 increases DSBs, we treated cells with MMS and examined the levels of γH2AX by western blotting analysis and fluorescence confocal microscopy. The HLTF-, UBC13-, BARD1-, and PARP1-depleted cells exhibited higher levels of γH2AX compared with control cells after treatment with MMS (Fig. 5a). Consistent with the western blot results, the HLTF-, UBC13-, BARD1-, and PARP1-depleted cells also exhibited stronger intensities of γH2AX foci compared with the control cells after treatment with the same concentrations of MMS (Fig. 5b, c). Similar results were also found in HLTF-KO and PARP1-KO HONE6 cells (Supplementary Fig. S4a–d). Since 53BP1 is enriched at DSBs, we also found that γH2AX foci were highly correlated with 53BP1 foci following MMS treatment (Supplementary Fig. S5a–c). Therefore, we conclude that more DSBs are generated in HLTF-, UBC13-, PARP1-, and BARD1-depleted cells, and this could contribute to the short replication tracks observed in these gene-deficient cells.

### Depletion of HLTF, BRCA1, and BARD1 reduces the level of RAD51 at stalled forks

**Fig. 6:**
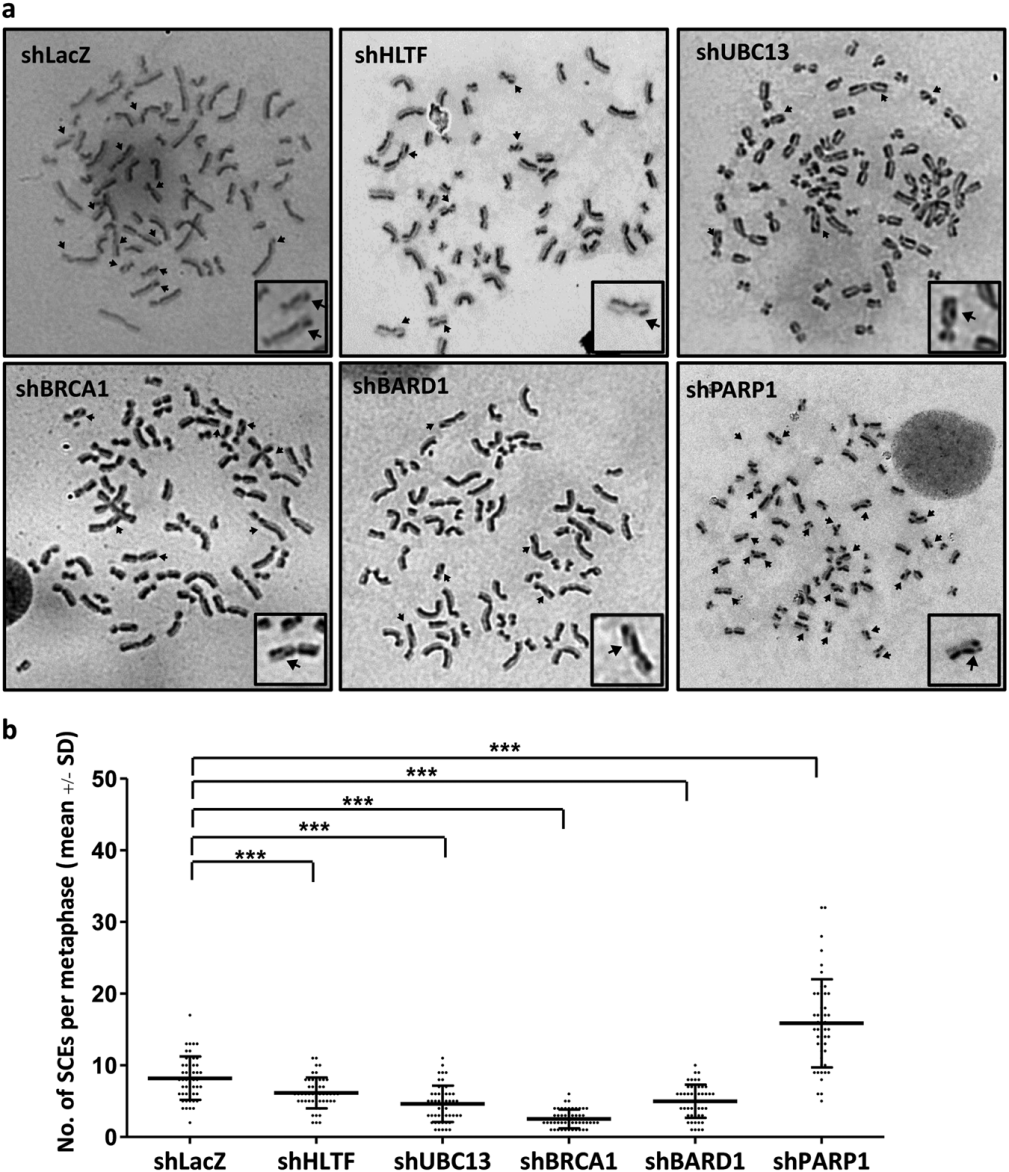
Sister chromatid exchange in HLTF-, PARP1-, BARD1-, and UBC13-depleted HONE6 cells. **a** The SCE analysis of the control cells (shLacZ) and the HLTF-, PARP1-, BARD1-, UBC13-, and BRCA1-depleted cells. **b** SCE was scored in 50 metaphases of each cell line. The asterisk *** represents *p* < 0.001. The *p* values were measured by the Mann–Whitney test.

SCE occurs during DNA replication, and it is an outcome of chromosome breaks and successful HR repair in a sequential manner. To test whether more chromosomal breaks during DNA replication, we examined the levels of SCEs in the HLTF-, PARP1-, and BARD1-depleted cells. DNA lesions can be induced by endogenous reactive oxygen species (ROS) within cells. As expected, the PARP1-depleted cells exhibited a high frequency of SCE compared with the shLacZ control cells (Fig. 6a, b). It is due to the fact that loss of PARP1 fails to repair SSBs induced by endogenous ROS, and as a result, more chromosome breaks are generated in the PARP1-depleted cells, resulting in a high frequency of SCE. However, the HLTF-, UBC13-, BRCA1-, and BARD1-depleted cells exhibited reduced frequency of SCE (Figs. 6a, b and S6a–c). As SCE is the result of chromosome breaks and HR repair during DNA replication, the reduced levels of SCEs could also attribute to less efficient HR repair in the HLTF-, UBC13-, BRCA1-, or BARD1-depleted cells. Therefore, we accessed the levels of RAD51 onto the damaged replication forks by using the EdU-PLA assay in the HLTF-KO and BRCA1- or BARD-depleted cells. In untreated cells, the number of cells with PLA foci and the foci number of PLA foci were similar between wild-type and gene-depleted cells (Supplementary Fig. S7a–g). After MMS treatment, we found that the number of cells with PLA foci and foci number was significantly reduced in the HLTF-KO and BRCA1- and BARD1-depleted cells. In contrast, the levels of RAD51-EdU-PLA foci were greatly increased in the PARP1-KO cells (Supplementary Fig. S7a–d). Additionally, by using confocal microscopy, we also observed that the levels of RAD51 increased over time following MMS treatment in wild-type and PARP1-KO cells (Supplementary Fig. S7h, i, k). In contrast, the levels of RAD51 foci significantly reduced in the HLTF-KO cells following MMS treatment (Supplementary Fig. S7h, j, k). These results were consistent with the RAD51/EdU-PLA assay. Therefore, we conclude that the reduced levels of SCE in the HLTF-, BRCA1-, and BARD1-depleted cells is due to reduced levels of RAD51 at damaged forks, thus reducing the efficiency of HR repair.

### The depletion of HLTF, UBC13, BARD1, and PARP1 sensitizes cells to MMS treatment

To determine whether the depletion of HLTF, UBC13, BARD1, and PARP1 sensitizes cells to MMS, we performed cytotoxicity and colony formation assays in the HLTF-, UBC13-, BARD1-, and PARP1-depleted cells. We treated these cells with various concentrations of MMS. Compared with the control shLacZ cells, the HLTF-, UBC13-, BARD1-, and PARP1-depleted cells were sensitized to MMS, as revealed by an MTT assay (Supplementary Fig. S8b). More importantly, chronic low-dose treatment with MMS for 10 days also sensitized cells to MMS as revealed by a colony formation assay (Supplementary Figs. S8a and S9a, b). Similar results were also found in HLTF-KO and PARP1-KO HONE6 and bladder cancer T24 cells (Supplementary Figs. S8c, d and S10a–c).

## Discussion

In this study, we identify a novel protein–protein interaction between HLTF and PARP1. The interaction reinforces each other to damaged forks and facilitates the progression and stability of damaged forks from collapse into DSBs.

By using confocal microscopy, we found that HLTF localizes both in cytoplasm and nucleus, while PARP1 localizes only in nucleus (Supplementary Fig. S11). Therefore, we think the HLTF/PARP1 complex resides in nucleus. The interaction does not increase protein stability of one and the other, since the protein turnover rates are similar among wild-type, HLTF-KO, and PARP1-KO cells (Supplementary Fig. S2). We believe that these interactions are independent of poly-ADP-ribosylation, since MMS and olaparib (PARP1 inhibitor) treatment did not affect the strength of interaction (Supplementary Fig. S12). Additionally, the GST fusion proteins isolated from bacteria *E. coli Rosetta* still interact with PARP1 and BARD1, and bacteria lacks the poly-ADP-ribosylation in cells.

The PARP1–BARD1 interaction is enhanced by the methylating agent MMS, but not by UV or cisplatin treatment. It could be due to the fact that UV-induced cyclobutane pyrimidine dimers and pyrimidine–pyrimidine (6–4) photoproducts can be largely removed by nucleotide excision repair^47,48^. The remaining photoproducts are efficiently bypassed by TLS polymerase POLH^49–51^. Cisplatin-induced crosslinks can be repaired by the Fanconi anemia pathway and can be also bypassed by the TLS polymerase POLH^52^. These activities compensate the need of TS and HR pathway. However, when cells are treated with enormous dose of MMS ranging from 0.05% (6 mM) to 1% (118 mM) of MMS, which overwhelm the repair capacity of BER and TLS POLK^53,54^. As a result, TS and HR pathways are recruited to repair these damaged forks.

We found that MMS treatment induces PCNA ubiquitination over time (Supplementary Fig. S13a, b). However, the levels of PCNA monoubiquitination and polyubiquitination are significantly reduced in the HLTF-KO and PARP1-KO cells. The results from HLTF-KO cells are consistent with previous publication^9^. The reduced levels of PCNA ubiquitination indicate that bypassing capacity by TLS and TS may be compromised in the HLTF-KO and PARP1-KO cells. It could lead to more DSBs in the HLTF- and PARP1-deficient cells. Indeed, our data support this notion, which shows higher levels of γH2AX and MMS sensitivity in the HLTF- and PARP1-depleted cells (Figs. 5, 6, S4, S8, and S10).

Our DNA fiber analysis revealed that the depletion of HLTF, PARP1, and BARD1 further reduces replication tracks and concomitantly increases the number of stalled forks after MMS treatment. It suggests that HLTF, PARP1, and BARD1 can assist in replication progression past DNA lesions, therefore preventing the accumulation of stalled forks. In support of the PARP1 function in the TS pathway, several recent reports show that PARP1 is involved in the formation of reversed forks^20,55,56^.

A recent paper from the Cimprich lab shows that HLTF contributes to replication slowing in response to replication stress^18^. In their experiment, 50 μM of HU was used to induce replication stress. It seems that their results are contradictory to our DNA fiber analysis. However, if one considers the types of chemicals to induce replication stress, the differences between our results and their published results could be due to the differences in types of replication stress. HU causes replication stress by limiting dNTP pools, however, DNA is not damaged during the 30-min treatment^57^. Different from HU-induced replication stress, MMS causes DNA lesions by DNA alkylation without depleting dNTP pools. In that sense, cells have sufficient dNTPs to replicate DNA in the presence of MMS, however, DNA is damaged. Therefore, the shorter replication tracks in the HLTF-depleted cells could be specific to MMS treatment. Indeed, a previous paper from the Haracska lab also supports our results^14^.

Previous studies have shown that MMS causes SSBs on a DNA template during the S-phase of cell cycle. N-methylpurine DNA glycosylase is responsible for the removal of methylated purines, therefore generating SSBs^36^. Therefore, the short replication tracks shown in the HLTF-depleted cells could be due to the fact that DNA replication encounters more frequent SSB templates in the HLTF-depleted cells. As a result, DNA replication terminates due to encountering those broken templates in the HLTF-deficient cells. In support of this notion, we observed that the levels of PARP1 at damaged forks are reduced in the HLTF-KO cells (Fig. 3). Given that PARP1 is involved in SSB repair, the reduced levels of PARP1 at damaged forks could result in more SSBs generated in cells. Indeed, the HLTF-depleted cells show higher levels of γH2AX than the wild-type cells, which supports our explanation. In contrast, in wild-type cells, HLTF and PARP1 reinforce with each other to damaged forks, therefore reducing the levels of SSBs. Additionally, HLTF promotes the fork reversal structure, which converts the damaged forks into reversed forks. As a result, DNA replication can be restarted by using the undamaged sister chromatid as a template, which resulting in longer replication tracks in wild-type cells than in HLTF-depleted cells.

The importance of TLS and TS is to prevent prolonged stalling of replication forks and DSBs associated with fork collapse in response to replication stress^4^. However, cells encounter large amount of DNA lesions, which overwhelm the repair capacity of BER and also the bypass capacity of TLS and TS pathways. As a result, the damaged forks are collapsed into DSBs. In conclusion, we propose a model in which PARP1 and HR have distinct early and late roles during replication stress: one is to facilitate fork reversal in collaboration with HLTF when forks are still viable; the other is to repair broken forks through HR when damaged forks have collapsed (Supplementary Fig. S14).

## Materials and methods

### Cell culture

The nasopharyngeal carcinoma cell line HONE6 was maintained as described previously^58,59^. The human embryonic kidney cell line HEK293T was maintained as described previously^9,10^.

The detailed “Materials and Methods” are described in Supplementary Material and Methods (Supplementary Fig. S15 and Tables S2 and S3).

## Supplementary information

supplementary materials and methods

supplementary figure and table legends

supplementary Figure S1

supplementary Fig S2

supplementary Figure S3

supplementary Figure S4

supplementary Figure S5

supplementary Figure S6

supplementary Figure S7

supplementary Figure S8

supplementary Figure S9

supplementary Figure S10

supplementary Figure S11

supplementary Figure S12

supplementary Figure S13

Supplementary Figure S14

supplementary Table S1

supplementary Table S2

supplementary Table S3
