# Supplementary material for: The HLTF–PARP1 interaction in the progression and stability of damaged replication forks caused by methyl methanesulfonate
Source: Oncogenesis. 2020 Dec 7;9(12):104. doi: 10.1038/s41389-020-00289-5 (PMC7719709; doi:10.1038/s41389-020-00289-5)
Supplement: Supplementary file 1 — supplementary materials and methods [file 41389_2020_289_MOESM1_ESM.pdf]

## Supplementary Materials and Methods

### Cell culture

The nasopharyngeal carcinoma cell line HONE6 was maintained in RPMI medium supplemented with 5% fetal bovine serum, 1% glutamine, and 1% penicillin/streptomycin in a 37 °C incubator with 5% $CO_2$. The human embryonic kidney cell line HEK293T was maintained in DMEM medium supplemented with 10% fetal bovine serum, 1% glutamine, and 1% penicillin/streptomycin in a 37 °C incubator with 5% CO2. The bladder cancer cell line T24 (ATCC® HTB4™) was maintained in McCoy's 5a media supplemented with 10% fetal bovine serum, 1% glutamine, and 1% penicillin/streptomycin in a 37 °C incubator with 5% $CO_2$.

### Co-immunoprecipitation (CoIP)

Cells were treated with various doses of DNA-damaging treatments. Then, the cells were lysed in NETN-100 lysis buffer (0.5% Nonidet P-40, 100 mM NaCl, 50 mM Tris-HCl, pH8.0, 2 mM EDTA, pH8.0 and protease inhibitor cocktail (MD Biol)). The cell lysates were then sonicated to fragment the chromosomal DNA. Following centrifugation, the supernatant was transferred into a clear tube, and was then incubated with the anti-FLAG and anti-GFP antibodies at 4°C overnight. The next day, protein G-sepharose beads were added into the samples and incubated at 4°C for 2 hours. After being washed with NETN-100 lysis buffer 3 times, the immunoprecipitates were mixed with 4x Laemmli sample buffer and then boiled for 10 minutes. Finally, the samples were resolved by a SDS-PAGE, followed by Western blotting analysis.

**RNA interference**

The shRNA packed lentivirus was generated by the transfection of HEK293T cells with packing plasmids, pCMVΔR8.91 and pMD.G, and pLKO_TRC005 vector containing control shLacZ, shHLTF, shHLTF #2, shPARP1, shPARP1 #2, shBARD1, shBARD1 #2, shUBC13, shUBC13 #2, or shBRCA1 shRNAs. Sequences of shRNAs are listed in Table S2. HONE6 cells were infected with these shRNA packed lentiviruses followed by selection with 2 μg/ml puromycin for one week. The depletion of genes was verified both by qRT-PCR and by Western blotting. All RNAi reagents were obtained from the National RNAi Core Facility, Academia Sinica, Taiwan.

**The generation of HLTF and PARP1 knockout HONE6 clones by CRISPR-Cas9**

HLTF- and PARP1-knockout (KO) HONE6 cells were generated by CRISPR/Cas9 gene knockout strategy using a pLAS2w-lentiCRISPR (P2A) plasmid. The single-guide RNAs (sgRNA) targeting HLTF and PARP1 were designed from the online tool CRISPR DESIGN (http://crispr.mit.edu/). The sgRNA sequence targeting HLTF is 5'-GGT TGG ACT ACG CTA TTA CA-3', and the sgRNA sequence targeting PARP1 is 5'-GAG TCG AGT ACG CCA AGA GC-3'. The sgRNA fragments were ligated into *Bsm*BI sites of pLAS2w-lentiCRISPR (P2A) plasmid. The resulting pLAS2w-lentiCRISPR plasmids were transfected into HEK293T cells using TransIT®-LT1 Reagent (Mirus) to generate lentivirus. HONE6 cells were then infected with the lentivirus and selected with 1 μg/ml puromycin for 10 days. Approximately 20 colonies of each knockout cell line were selected for Western blotting analysis. The deletion of HLTF and PARP1 was also confirmed by genomic sequencing.

**EdU-PLA assay**

HONE6 cells were plated in 4-well chamber slides (Millipore) at a density of 2 x $10^4$ and pulse-labeled with 10 μM EdU for 10 minutes followed by treatment with 1.2 mM (0.01%) MMS for 1- and 3-hr. After that, the cytoplasmic fractions of the cells were removed by extraction buffer (25 mM HEPES, pH7.4, 50 mM NaCl, 3 mM $MgCl_2$,1 mM EDTA, 0.3M Sucrose, 0.5% Triton X-100). Then the cells were fixed with 3.5 % paraformaldehyde in PBS for 30 minutes at room temperature. The Click reaction was performed with biotin-azide for 30 minutes and then quenched with PBS containing 10% FBS and 0.1% Triton X-100 for 30 minutes. Two primary antibodies against biotin and target proteins were used to detect nascent synthesized DNA and the target proteins, respectively. The primary antibodies used in this study are listed in Table S3. Two oligonucleotide-conjugated secondary antibodies, anti-rabbit PLUS and anti-mouse MINUS antibodies (Duolink, Sigma-Aldrich), were applied to samples and incubated for 60 minutes at 37°C. Ligases and DNA polymerases were then applied to the samples according to the manufacturer's protocol (Duolink, Sigma-Aldrich). Finally, the samples were mounted with Duolink In Situ Mounting Medium with DAPI for 15 minutes at room temperature and analyzed using a Nikon eclipse 80i microscope equipped with a Plan Fluor 40x/0.75 DIC M/N2 objective. The resulting images were then measured using NIS Elements D4.20.00 software (Nikon).

**Plasmid construction**

The wild-type human HLTF gene was amplified by PCR with primers and cloned into the *Hind*III and *Bag*II sites of p3xFLAG-CMV-10 [1]. All HLTF deletion mutants were generated by site-directed mutagenesis (Agilent Technologies) with

primers and the plasmid containing the FLAG-HLTF gene as the template. The HIRAN-DEXDc (1-763) domain of HLTF was generated with two primers, 5’-GAC GAT GAC AAG CTT TCC TGG ATG-3’ and 5’-AAAAG ATCTG CCATT GGAAG ACACT GCATT TGT-3’. The DEXDc (180-731) domain of HLTF was generated with two primers, 5’-AAA AAG CTT TTC AAT TTG GAA AGT GGT TGG GGC-3’ and 5’-AAA AGA TCT GCC ATT GGA AGA CAC TGC ATT TGT-3’. The RING-HELICc (721-1009) domain of HLTF was generated with two primers, 5’- AAA AAG CTT CCT GTT TTG GAG TTA CCA GAA CGT-3’ and 5’-CGA CTG GTA CCG ATA TCA GAT CTC-3’. The human BARD1-turboGFP clone was purchased from the OriGene (Catalog No: RG211511). The human pCMV-sport 6-PARP1 clone was purchased from the OriGene (Catalog No: SC119157). The plasmid containing PARP1 was cloned into the *Sac*II and *Xho*I sites of pEGFP-N1. To generate the GST-HLTF fusion clones, the HIRAN and DEXDc domains of HLTF were cloned into the *Xma*I and *Xho*I sites of the plasmid pGex6p-1. All of the constructs were confirmed by DNA sequencing.

**Western blotting**

Cells were harvested in lysis buffer (50mM Tris, pH7.5, 150 mM NaCl, 1mM EDTA, 0.1% Triton X-100, protease inhibitor cocktail (MD Biol)). The lysates were then sonicated (30 sec ON and 30 sec OFF for 10 min, Bioruptor$^{TM}$, UCD-200, Diagenode), followed by the addition of Laemmli sample buffer (4% SDS, 125mM Tris (pH 6.8), 40% glycerol, 0.01% Bromophenol blue, 10% 2-Mercaptoethanol) and the boiling of samples for 5 minutes. The protein concentration was determined by the Hartree-Lowry protein assay. Samples were separated by a 6-15% SDS-PAGE gel and transferred onto PVDF membranes. The PVDF membranes were blocked

with 5% skim milk in TBST (20 mM Tris, pH 7.6, 150 mM NaCl, 0.1% Tween 20) for 1 hour at room temperature. Subsequently, the membranes were incubated with primary antibodies at room temperature for 1 hour, followed by incubation with the horseradish peroxidase (HRP)-conjugated secondary antibodies at room temperature for 1 hour. The primary antibodies used in this study are listed in Table S3. Images were detected by a chemiluminescence imaging system (GeneGnome 5 Bio Image, Syngene). Original blots are shown in supplementary Fig. S15.

**GST pulldown assay**

Glutathione S-transferase (GST) fusion proteins were expressed in *Escherichia coli Rosetta*. The *Rosetta* cells were mixed with 1mM IPTG to induce the expression of fusion proteins at 30°C for 2 hours. Then, the cells were resuspended with STE buffer (10mM Tris, pH 8.0, 1mM EDTA and 150mM NaCl), and with protease inhibitors (0.2M PMSF and 5mg/ml Leupeptin). The cells were then lysed by sonication (BioruptorTM, UCD-200, Diagenode) for 10 minutes (30 sec ON and 30 sec OFF) and then mixed with 10% Triton X100 to a final concentration of 1-2% of Triton X100. The samples were rotated at 4°C for 30 minutes and centrifuged at 12000 rpm at 4°C for 10 minutes. The resulting supernatants were mixed with glutathione (GSH) beads and then rotated at 4°C overnight. The GST fusion proteins were purified by washing them with PBS 3 times. The purified GST fusion proteins were then incubated with cell lysates derived from HEK293T cells at 4°C for 3 hours. After being washed with wash buffer (50mM Tris, pH7.5, 150 mM NaCl, 1mM EDTA, 0.1% Triton X-100), the pull-down complex was mixed with 4x

Laemmli sample buffer and boiled for 10 minutes. The proteins associated in the complex were separated by a SDS-PAGE and analyzed by Western blotting.

**DNA fiber analysis**

HONE6 cells were cultured in a 100-mm culture dish at a density 2 x $10^6$ for 16 hours and pulse-labeled with 25 μM CldU for 20 minutes, followed by treatment with MMS or UV and simultaneously pulse-labeling with 250 μM IdU for 30 minutes. The cells were then harvested and their nuclear fractions were isolated with buffer A (10 mM HEPES, pH 7.9, 10 mM KCl, 1.5 mM MgCl2, 0.34 M sucrose, 10% glycerol). 2 μl samples of nuclear fractions were then plated onto glass slides and lysed with spreading buffer (0.5% SDS, 50mM EDTA, 200mM Tris, pH 5.5) for 4 minutes. For each slide, a coverslip was placed on the area of the spreading buffer, which was allowed to slide down along the slide, resulting in the spreading of DNA fibers along the slide. The DNA fibers were fixed in a 3:1 methanol/acetic acid buffer for 10 minutes, denatured with 2N HCl for 1 hour at room temperature, and then blocked in PBS buffer (1% BSA, 0.1% Tween20 in PBS). The rat anti-BrdU primary antibody (1:200, abcam, ab6353) against CldU and the mouse anti-BrdU primary antibody (1:200, BD Biosciences, 347580) against IdU were added on the slides and incubated at 4°C overnight. The goat anti-rat AlexaFluor-594 secondary antibody (1:500, Thermo Fisher Scientific, 1301853) and the anti-mouse AlexaFluor-488 secondary antibody (1:500, Thermo Fisher Scientific, 1613346) were added on the slides for 90 minutes at room temperature. Images were acquired by a Nikon eclipse 80i microscope equipped with a Nikon Plan Apo 100x/1.40 Oil DIC objective. At least 200 fibers of each sample were analyzed by using NIS Elements D4.20.00

software (Nikon), and graphs were plotted with GraphPad Prism software (Version 5.0).

**Immunofluorescence microscopy**

Cells were cultured at a density of 3.5 x $10^4$ in four-well chamber slides and chronically treated with 0.001% or 0.0015% MMS for 24 hours. Then, the cells were fixed with 3.5% paraformaldehyde for 15 minutes and permeabilized with 2% Triton X-100 in PBS for 10 minutes at room temperature. The fixed cells were then incubated in blocking buffer (10% FBS, 0.1% Triton X100 in PBS) for 1 hour at room temperature and further incubated with anti-γH2AX antibody (1:200, Millipore, 05-636) at 4℃ overnight. Alexa Fluor 594-labeled goat anti-mouse IgG (H+L) antibody (1:500, Thermo Fisher Scientific, A-11032) was then used to detect γH2AX foci. ProLong Gold Antifade Mountant with DAPI (Thermo Fisher Scientific, P36935) was used to stain nuclei. Images were captured using a Nikon TE-2000 confocal microscope. 150 nuclei per treatment were used to quantify the intensity of the γH2AX foci using FV10-ASW software (Olympus Life Science)

**Sister chromatid exchange (SCE)**

HONE6 cells were seeded at a density of 2 x $10^6$ in a 100-mm culture dish and incubated with 9 μg/mL 5-bromodeoxyuridine (BrdU) (Sigma-Aldrich) for 46 hours. Then, the cells were treated with 0.1 μg/ml colcemid (Thermo Fischer Scientific, 15212012) for 1 hour to harvest metaphase chromosomes. Subsequently, the cells were trypsinized and incubated in 75 mM KCl at 37℃ for 10 minutes, followed by

the addition of fixing solution (methanol/acetic acid, 3:1). The fixed cells were then dropped onto slides to spread the chromosomes. The slides were incubated with 50 μg/ml Hochest 33258 (Thermo Fisher Scientific, H1398) for 10 minutes at room temperature, followed by crosslinking with 1200 J/m$^2$ of UV irradiation in saline-sodium citrate (SSC). Finally, the slides were stained in 4% Giemsa in Gurr's buffer for 4 minutes, rinsed with deionized water twice, and dried at room temperature. Images of metaphase chromosomes were acquired by a Nikon eclipse 80i fitted with Plan Fluor 60x/0,50-1,25 Oil Iris DIC N2 objective and NIS Elements D4.20.00. For each cell line, 50 metaphases were analyzed to determine the number of sister chromatid exchanges.

**Colony formation assay**

Cells were plated at a density of 1 x 10$^4$ into 100-mm culture dishes in duplicate and chronically treated with cisplatin, methyl methanesulfonate (MMS), or 4-nitroquinoline-1-oxide (4NQO) at the indicated concentrations. After incubation for 10 days, colonies were formed and stained with 1% crystal violet (in 25% methanol) for 1 minute. The stained plates were gently washed with water and then dried in the air overnight. The colonies were then counted using the GeneTools software program (Syngene). The colony formation was determined by the number of colonies of each treatment divided by the number of cells of the untreated controls.

**Cytotoxicity assay**

HONE cells were seeded in a 96-well plate at a density of 3 x 10$^3$ per well in triplicate and treated with cisplatin, MMS, and 4NQO at the various concentrations. Then the cells were incubated for 96 hours. The cell cytotoxicity was assessed by

reaction with 0.5 mg/ml of 3-(4,5-dimethylthiazol-2yl)- 2,5-diphenyltetrazolium bromide (MTT, Sigma-Aldrich) at 37$^{o}$C for 4 hours. Since cells with active metabolism convert MTT into a purple formazan product, the converted dye was solubilized with 5% sodium dodecyl sulfate (SDS, Sigma-Aldrich). The samples were measured at a wavelength of 595 nm using a microplate reader. The cytotoxicity was determined by the absorbance reads of each treatment normalized to the untreated controls.

## References

1 Motegi, A. et al. Polyubiquitination of proliferating cell nuclear antigen by HLTF and SHPRH prevents genomic instability from stalled replication forks. *Proc. Natl Acad. Sci. USA* **105,** 12411-12416 (2008)..
