# Supplementary material for: The HLTF–PARP1 interaction in the progression and stability of damaged replication forks caused by methyl methanesulfonate
Source: Oncogenesis. 2020 Dec 7;9(12):104. doi: 10.1038/s41389-020-00289-5 (PMC7719709; doi:10.1038/s41389-020-00289-5)
Supplement: Supplementary file 2 — supplementary figure and table legends [file 41389_2020_289_MOESM2_ESM.docx]

**Supplementary figure and table legends**

**Figure S1.** HLTF, PARP1, and BARD1 interact with each other *in vivo*. **(a)** The schematic representation of the protein-protein interaction by the PLA assay. **(b)** The representative images of PLA assay. The PLA foci are shown in red and DNA is stained with DAPI (blue). Antibodies against HLTF, PARP1, and BARD1 are indicated on top of the image. Cells were treated with 1.2 mM MMS and samples were collected at 0-, 1-, and 3-hr time points following the treatment. **(c)** Distribution of PLA foci from each cell derived from (b). At least 200 cells from each condition were measured. ns: no significance; *** P<0.001 (Mann–Whitney test). **(d)** The percent stacked column graph derived from (c). The number of PLA foci from each cell was classified into four groups: 0, 1~5, 6~9 and 10~ foci. The percentage of each group is indicated in the plot.

**Figure S2.** The protein stability of HLTF and PARP1 in wild type, HLTF-KO, and PARP1-KO HONE6 cells. Cells were treated with 100 μg/ml of cycloheximide to inhibit protein translation. Cells were harvested cells at 4, 8, 12, and 24 hours following cycloheximide treatment. The HLTF and PARP1 protein levels were determined by western blotting with specific antibodies indicated. α-tubulin was used as the loading control. The protein turnover rate was determined by a ratio of HLTF or PARP1 to its α-tubulin and normalized by the ratio at 0-hr timepoint.

**Figure S3.** Representative images of replication tracks of LacZ (control) and the HLTF-, UBC13-, BARD1-, and PARP1-depleted HONE6 cells in mock and MMS-treated conditions.

**Figure S4.** HLTF-KO and PARP1-KO HONE6 cells show higher levels of γH2AX than wild type control cells. The confocal microscopy of γH2AX (red) derived from wild type, HLTF-KO, and PARP1-KO HONE6 cells in the untreated (a) and MMS-treated (b) conditions. Cells were treated with 0.18 mM of MMS for 24 hours. Cells were fixed and immunostained with γH2AX antibody. DNA was stained with DAPI (blue). **(c) (d)** The foci number of γH2AX from each cell was quantified. The number of γH2AX foci of each cell was classified into two groups: >10 (greater than 10 foci) and <10 foci (less than 10 foci). At least 100 cells from each cell line were quantified.

**Figure S5.** The confocal microscopy of γH2AX and 53BP1 derived from the HONE6 cells. **(a)** The representative images of confocal microscopy. γH2AX (red) and 53BP1 (green) were detected by specific antibodies against γH2AX and 53BP1, respectively. DNA was stained with DAPI (blue). Cells were untreated (mock) or treated with MMS as indicated. A line in nucleus is a cross section for analyzing γH2AX and 53BP1 intensity shown in **(b) (c).** The intensity of γH2AX and 53BP1 was quantified by using the FV10-ASW software.

**Figure S6.** The SCE analysis in HLTF-depleted HONE6 cells. **(a)** HLTF was depleted by shHLTF #2 (TRCN0000272562). The depletion of HLTF was verified by western blot analysis. **(b)** The representative images of SCE derived from control (shLacZ) and HLTF-depleted cells. **(c)** SCE was scored in 50 metaphases of each cell line. The asterisk *** represents p<0.001. The p-values were measured by the Mann–Whitney test.

**Figure S7.** The RAD51/EdU PLA foci at damaged forks in the HLTF-, BRCA1-, BARD1-, and PARP1-depleted HONE6 cells. **(a)** The immunostaining of HLTF and PARP1 in the HLTF-KO and PARP1-KO HONE6 cells and the immunostaining of BRCA1 in the BRCA1-depleted HONE6 cells. β-tubulin was used as the loading control. **(b) (e)** Representative images of RAD51/EdU PLA foci in wild type, HLTF-KO, PARP1-KO, BRCA1-knockdown, and BARD1-knockdown cells. Cells were collected at the 0- and 3-hr timepoints following MMS treatment. **(c) (f)** Distribution of RAD51/EdU PLA foci from each cell derived from (b) (e), respectively. At least 200 cells from each condition were measured. The p-value was determined by the Mann–Whitney test. ns: no significance; * P<0.05; ** P<0.01; *** P<0.001. **(d) (g)** The percent stacked column graph derived from (c) (f), respectively. **(h) (i) (j)** The confocal microscopy of RAD51 (green) in wild type, HLTF-KO, and PARP1-KO HONE6 cells. DNA was stained with DAPI (blue). Cells were treated with 0.18 mM of MMS for 3 hours. Cells were fixed and immunostained with RAD51 antibody. **(k)** The intensity of RAD51 in each cell was quantified using the FV10-ASW software. At least 100 cells from each cell line were quantified. *** P<0.001 (Mann–Whitney test).

**Figure S8.** Depletion of HLTF, UBC13, BARD1, and PARP1 sensitizes HONE6 cells to MMS. **(a)** The colony formation assay. Cells were chronically treated with MMS and incubated for 10 days. The resulting colonies were stained with 1% crystal violet. Relative viability was determined by the number of colonies from treated cells relative to the number of cells without any treatment. **(b)** Cytotoxicity to MMS was determined by the MTT assay. **(c)** The colony formation assay of HLTF-KO and PARP1-KO HONE6 cells. Cells were chronically treated with 0.06, 0.12, 0.18, and 0.24 mM MMS and incubated for 10 days and stained with 1% crystal violet. **(d)** The MTT assay of HLTF-KO and PARP1-KO cells. The p-value was determined by the student t-test. ns: no significance; * p<0.05; ** p<0.01; *** p<0.001. The colony formation assays have been repeated at least twice and the MTT experiments have been repeated for at least three times. Similar trends were observed from these repeats.

**Figure S9.** Representative images of gene-knockdown **(a)** and gene-knockout HONE6 cells **(b)** by colony formation assay. Cells were chronically treated with 0.06, 0.12, 0.18, and 0.24 mM of MMS and incubated for 10 days.

**Figure S10.** HLTF-KO and PARP1-KO T24 cells are more sensitive to MMS than wild type cells. **(a)** Representative images of the knockout cells by colony formation assay. **(b)** The HLTF-KO and PARP1-KO T24 cells were chronically treated with 0.06, 0.12, 0.18, and 0.24 mM MMS and incubated for 10 days and stained with 1% crystal violet. **(c)** Cytotoxicity of the HLTF-KO and PARP1-KO T24 cells to MMS was determined by the MTT assay. The p-value of each test is shown. ns: no significance; * p<0.05; ** p<0.01; *** p<0.001 (student t-test)

**Figure S11.** HLTF localizes both in cytoplasm and nucleus, and PARP1 localizes only in nucleus. The confocal microscopy of HLTF (green) and PARP1 (red) derived from HONE6 cells. DNA was stained with DAPI (blue). Cells were collected at 0-, 1-, and 3-hr time points following MMS treatment.

**Figure S12.** The PARP1-HLTF interaction in the presence of olaparib. Cells were treated with 10 μM Olaparib for 2 hours, followed by 1.2 mM MMS treatments for 3 hours. The endogenous PARP1 was immunoprecipitated with an anti-PARP1 antibody and the pulldown HLTF was detected by an anti-HLTF antibody. IgG was used as the negative control. Input represents 5% of total cell lysates.

**Figure S13.** The levels of PCNA ubiquitination are reduced in the HLTF-KO and PARP1-KO cells. Cells were treated with 1.2 mM MMS and samples were collected at the 0-, 1-, and 3-hr time points following the treatment. The nuclear fraction of cells was isolated, lysed, and subjected to western blotting with a specific anti-PCNA antibody. The short exposure (a) and long exposure (b) of the same blot were shown. Ub and Ub^N^ indicate mono- and polyubiquitinated species of PCNA, respectively.

**Figure S14.** Schematic model for the function of HLTF, PARP1, and HR proteins in the TS pathway. In response to replication stress caused by MMS, HLTF, PARP1, and HR proteins are recruited to stalled forks, where they promote the formation of reversed forks. Replication restart could occur by using undamaged sister chromatids as a template through the reversed fork structure. MPG is also involved in the generation of SSB. The SSB results in SCE. However, in the absence of HLTF or PARP1, replication encounters more SSBs, and as a result replication tracks become shorter and increases the levels of γH2AX and SCE.

**Table S1.** The list of proteins identified in mass spectrometry.

**Table S2.** The list of targeting sequences of shRNA used in this study. All RNAi reagents were obtained from the National RNAi Core Facility, Academia Sinica, Taiwan.

**Table S3.** The list of antibodies used in this study.
