# Supplementary figures and images for: The HLTF–PARP1 interaction in the progression and stability of damaged replication forks caused by methyl methanesulfonate
Source: Oncogenesis. 2020 Dec 7;9(12):104. doi: 10.1038/s41389-020-00289-5 (PMC7719709; doi:10.1038/s41389-020-00289-5)

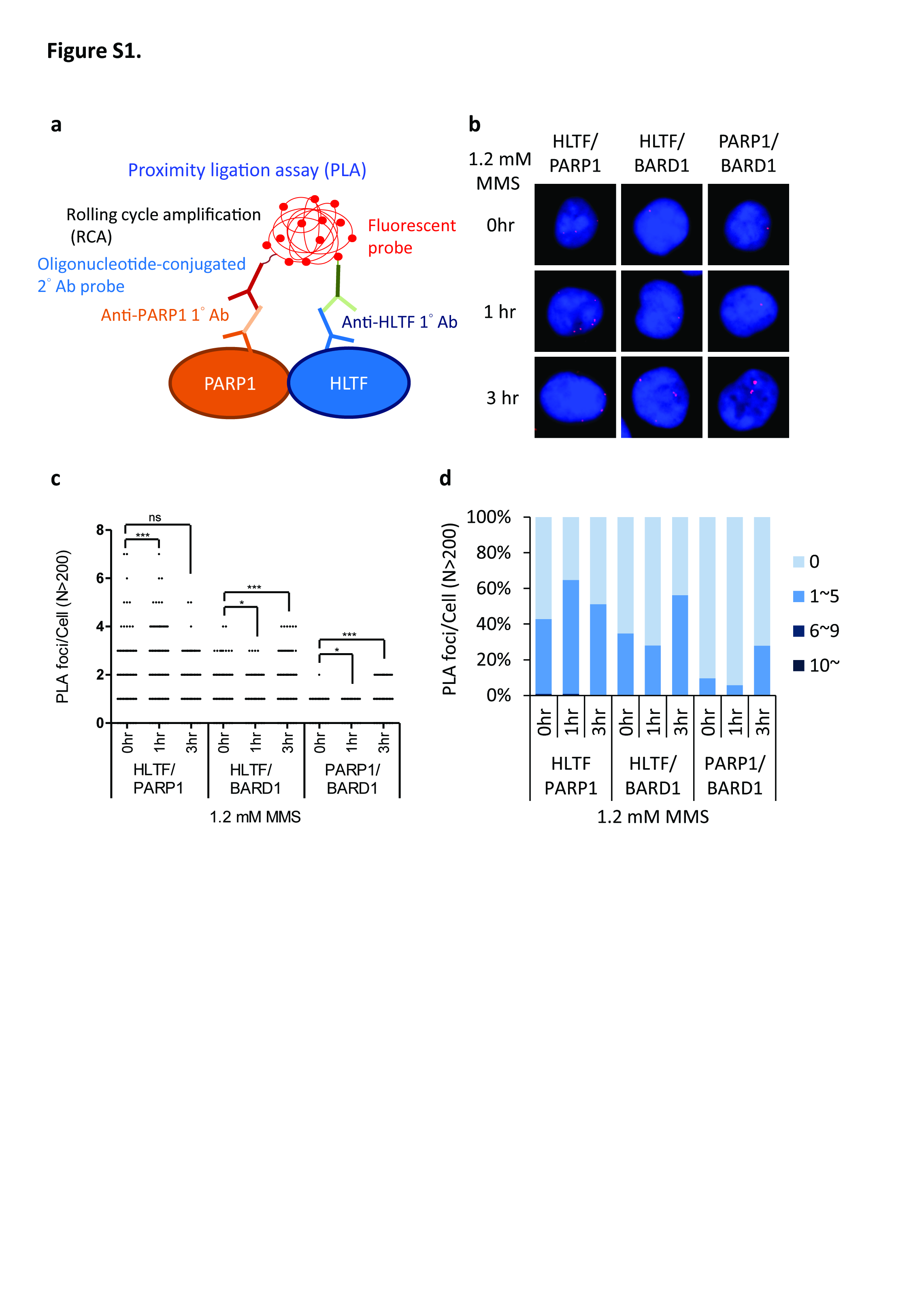

Supplement: Supplementary file 3 — supplementary Figure S1 [file 41389_2020_289_MOESM3_ESM.tif]

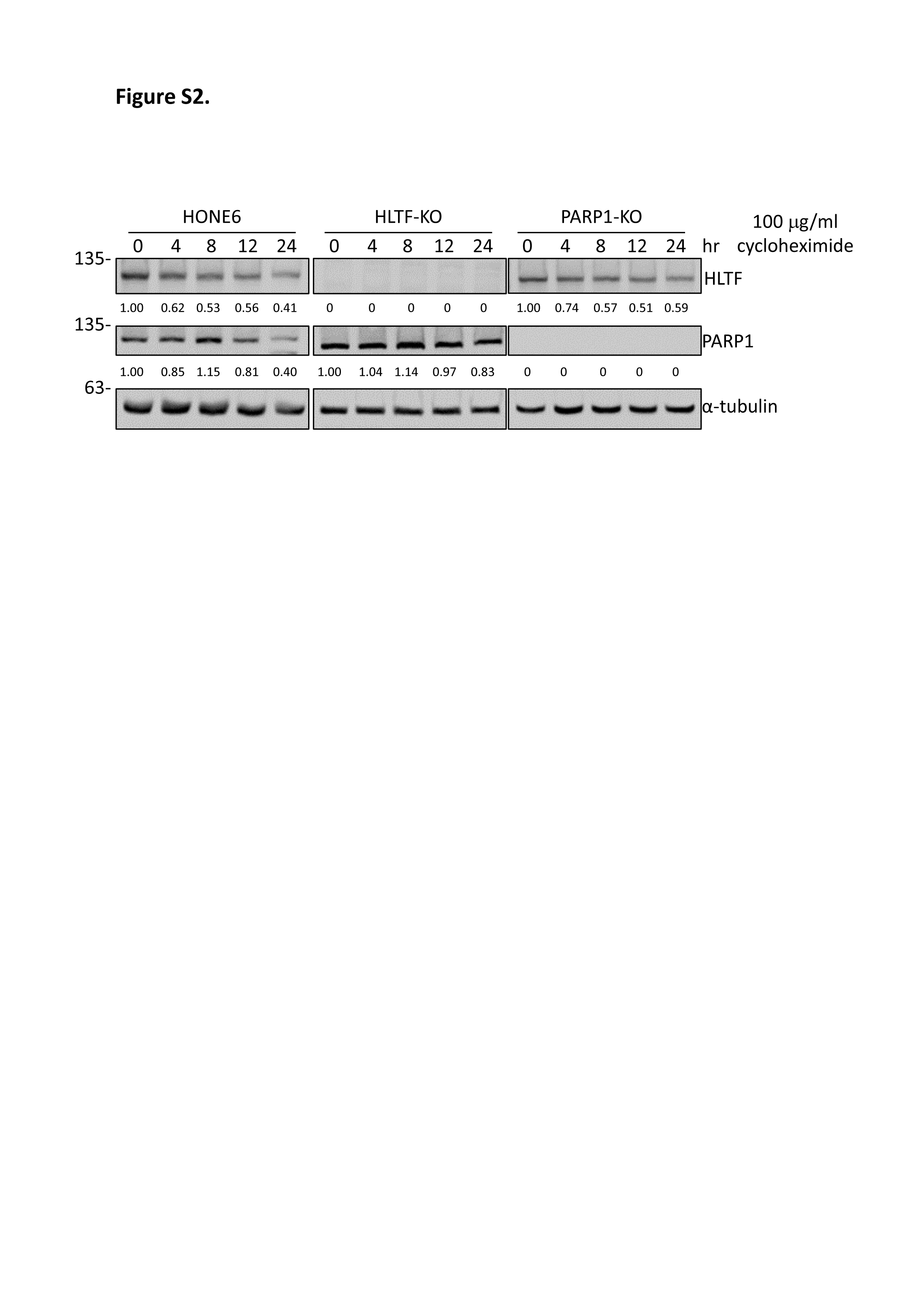

Supplement: Supplementary file 4 — supplementary Fig S2 [file 41389_2020_289_MOESM4_ESM.tif]

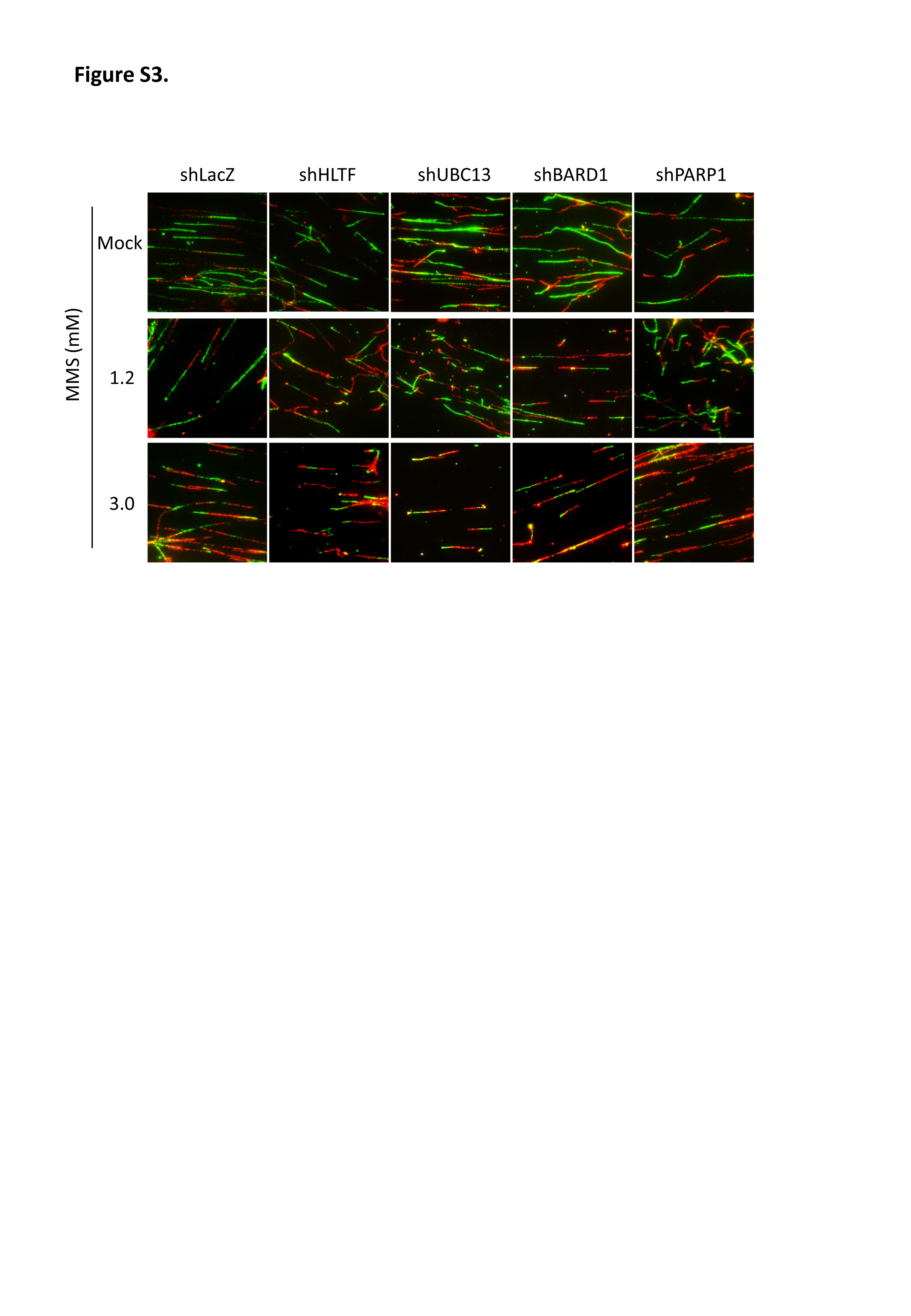

Supplement: Supplementary file 5 — supplementary Figure S3 [file 41389_2020_289_MOESM5_ESM.tif]

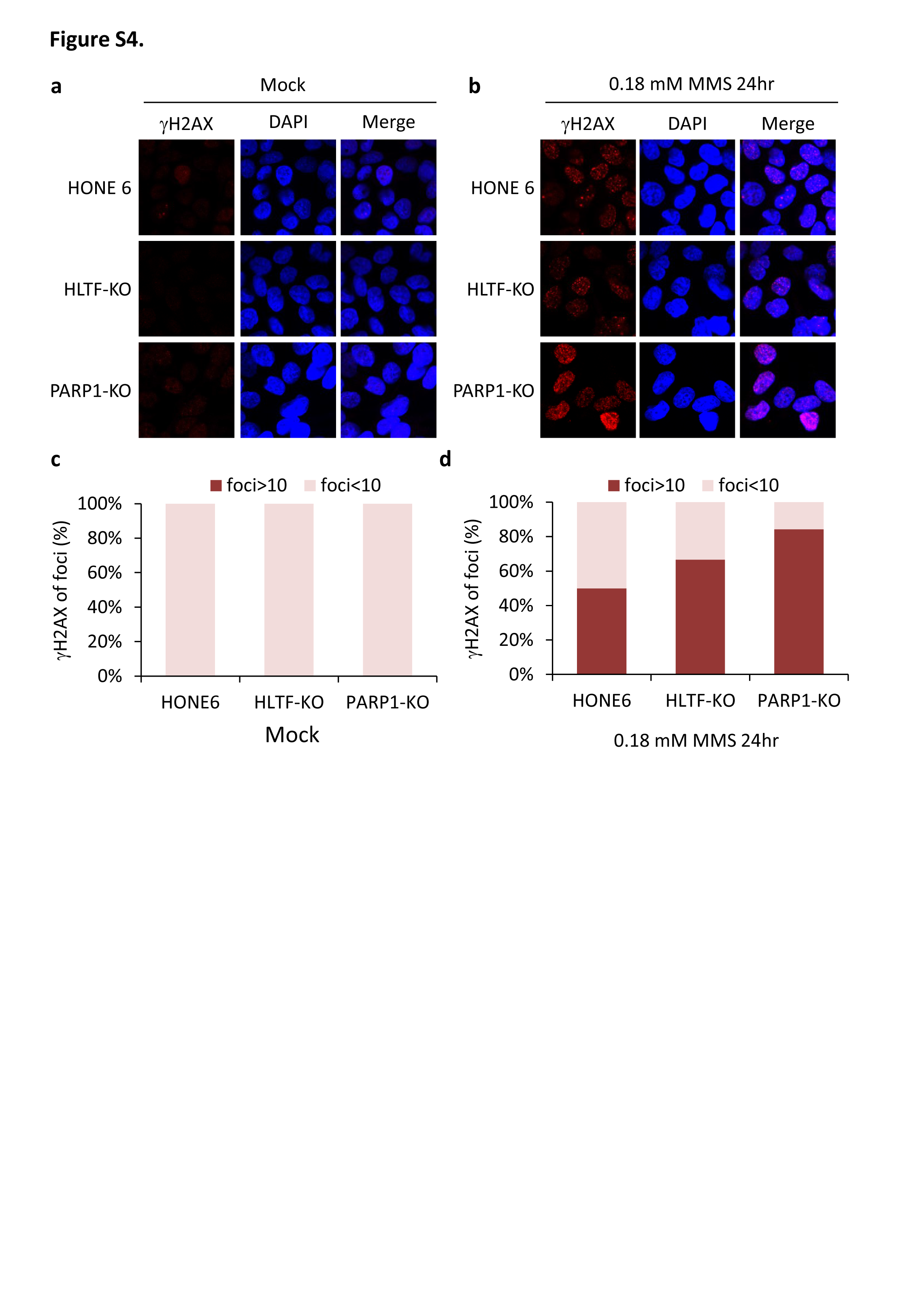

Supplement: Supplementary file 6 — supplementary Figure S4 [file 41389_2020_289_MOESM6_ESM.tif]

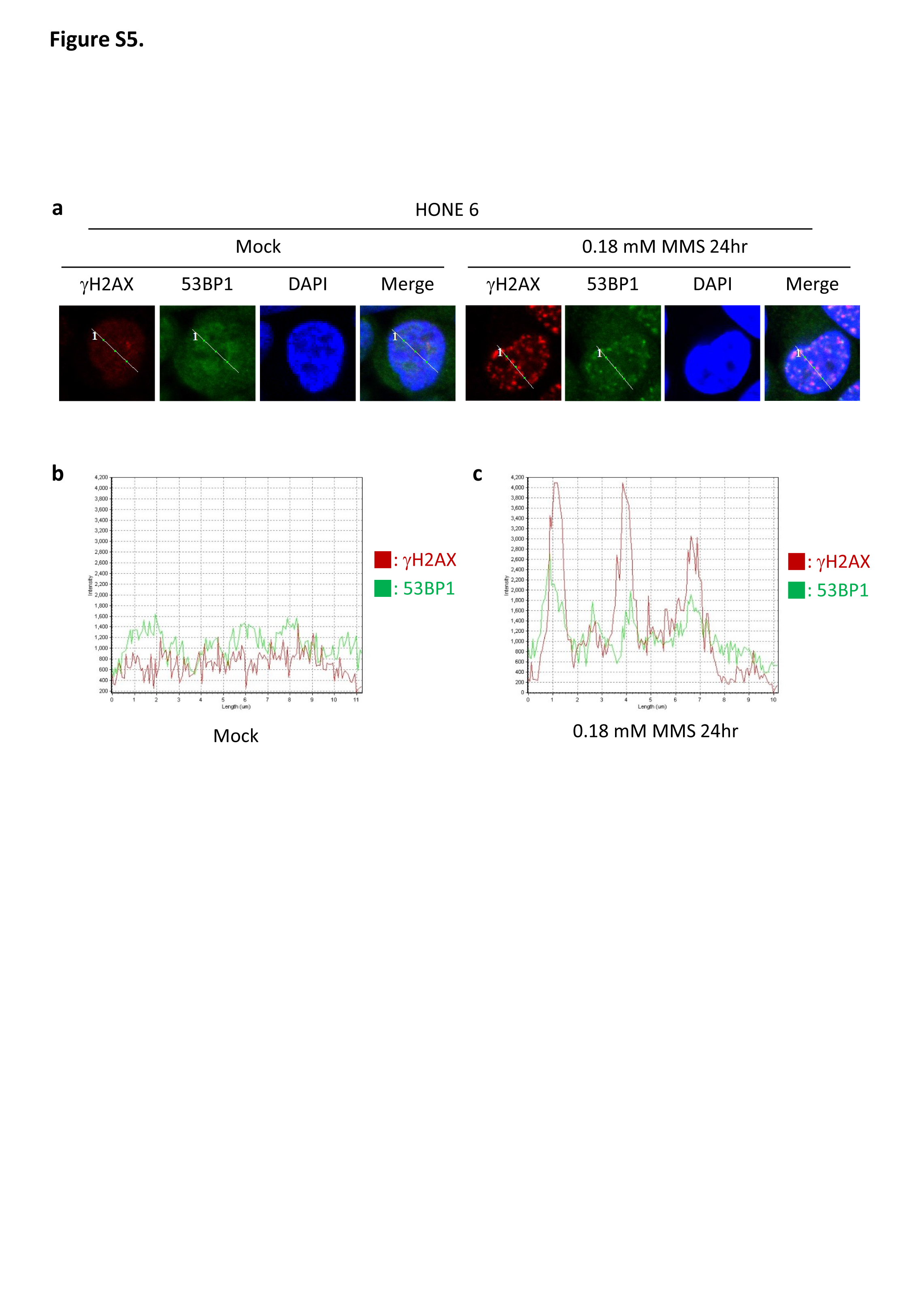

Supplement: Supplementary file 7 — supplementary Figure S5 [file 41389_2020_289_MOESM7_ESM.tif]

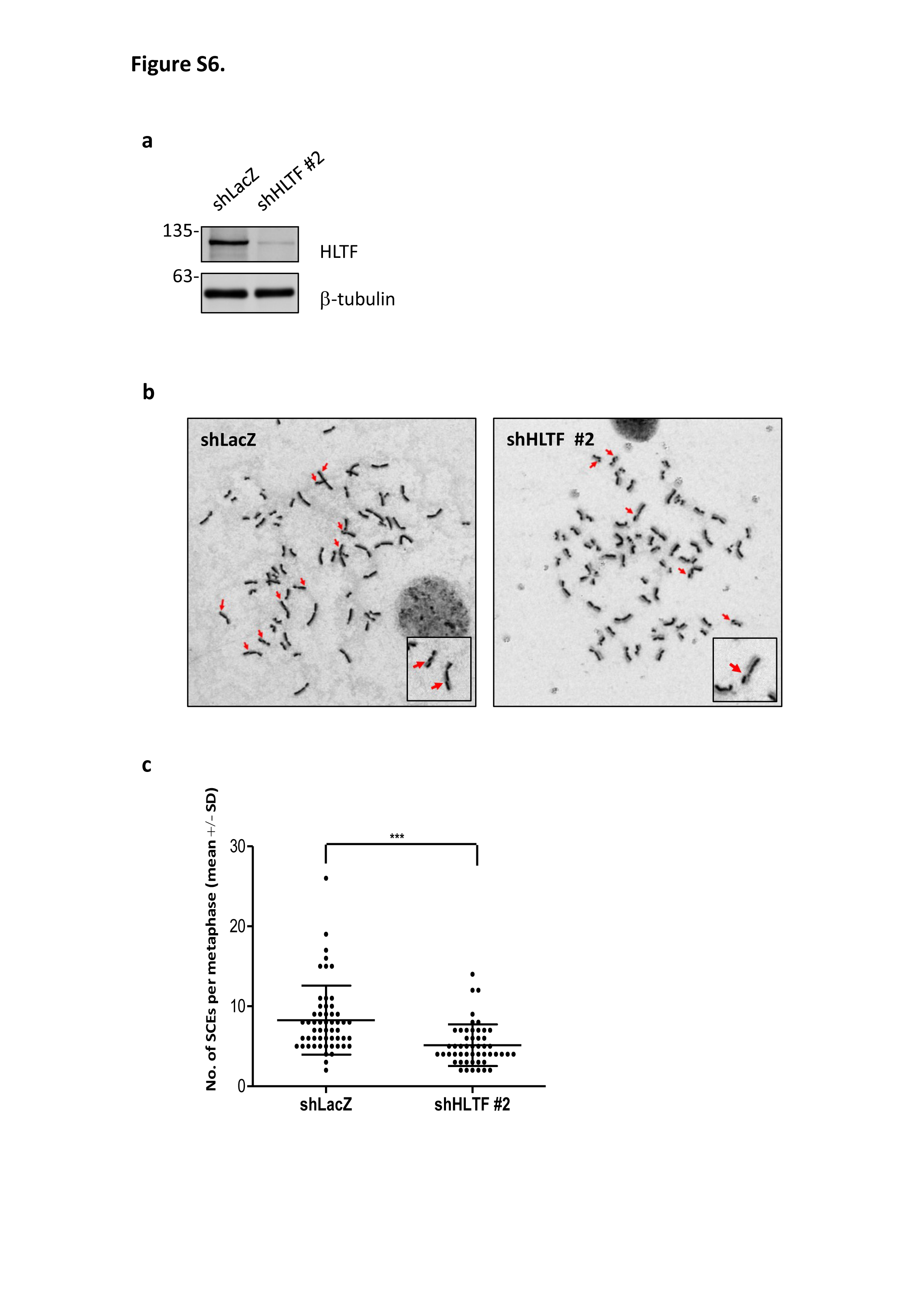

Supplement: Supplementary file 8 — supplementary Figure S6 [file 41389_2020_289_MOESM8_ESM.tif]

**Figure S7.**

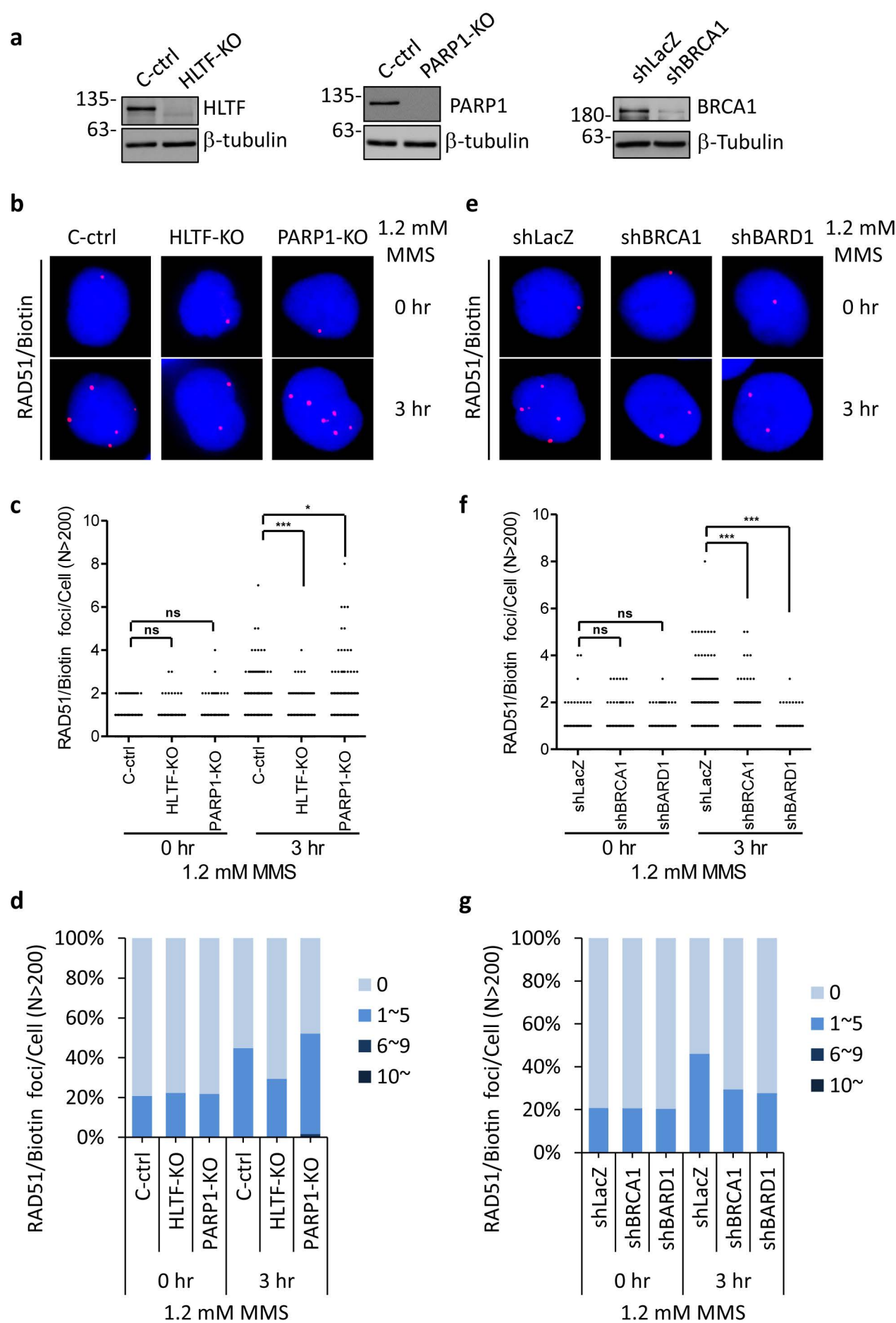

**Figure S7.**

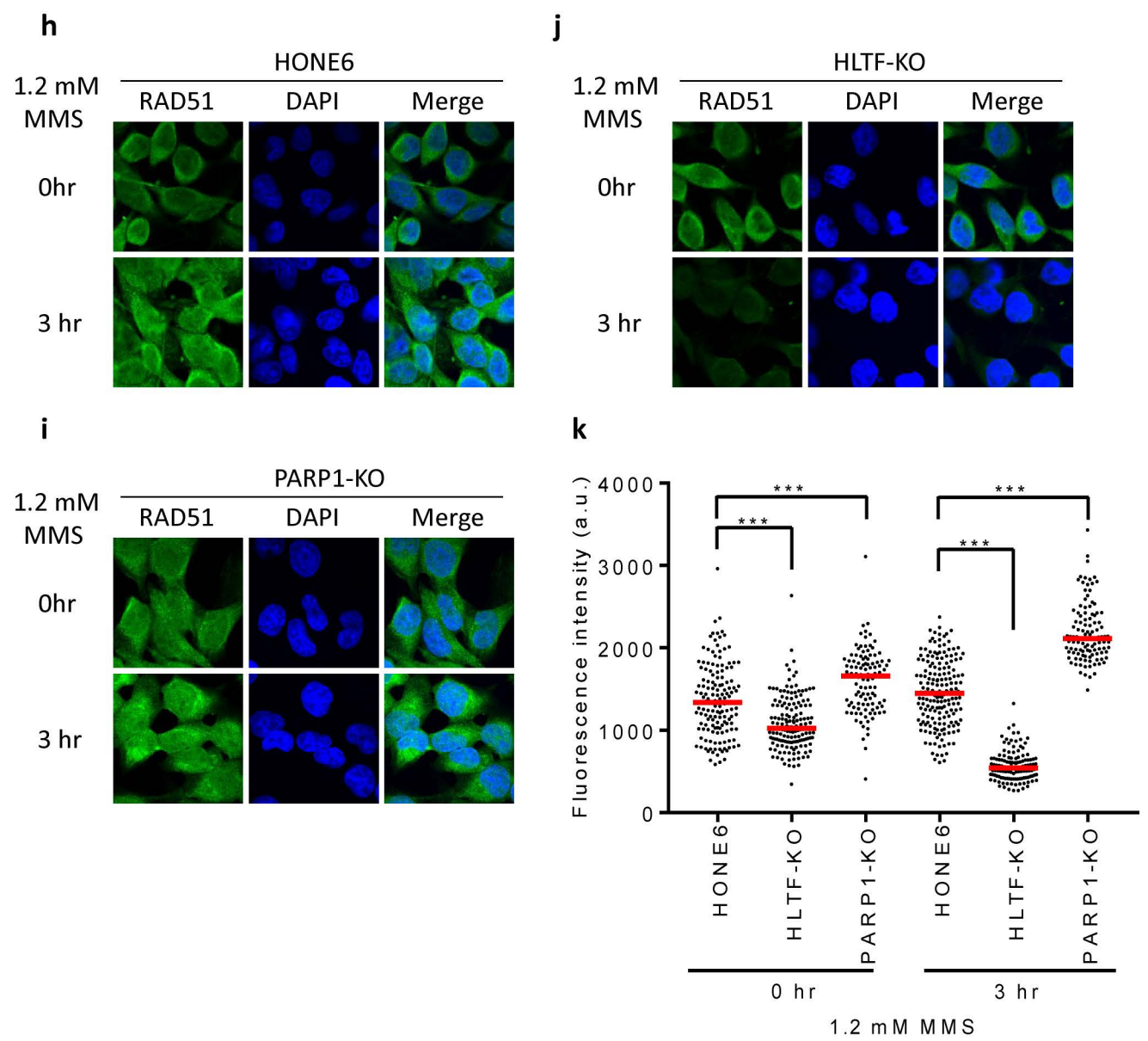

Supplement: Supplementary file 9 — supplementary Figure S7 [file 41389_2020_289_MOESM9_ESM.pdf]

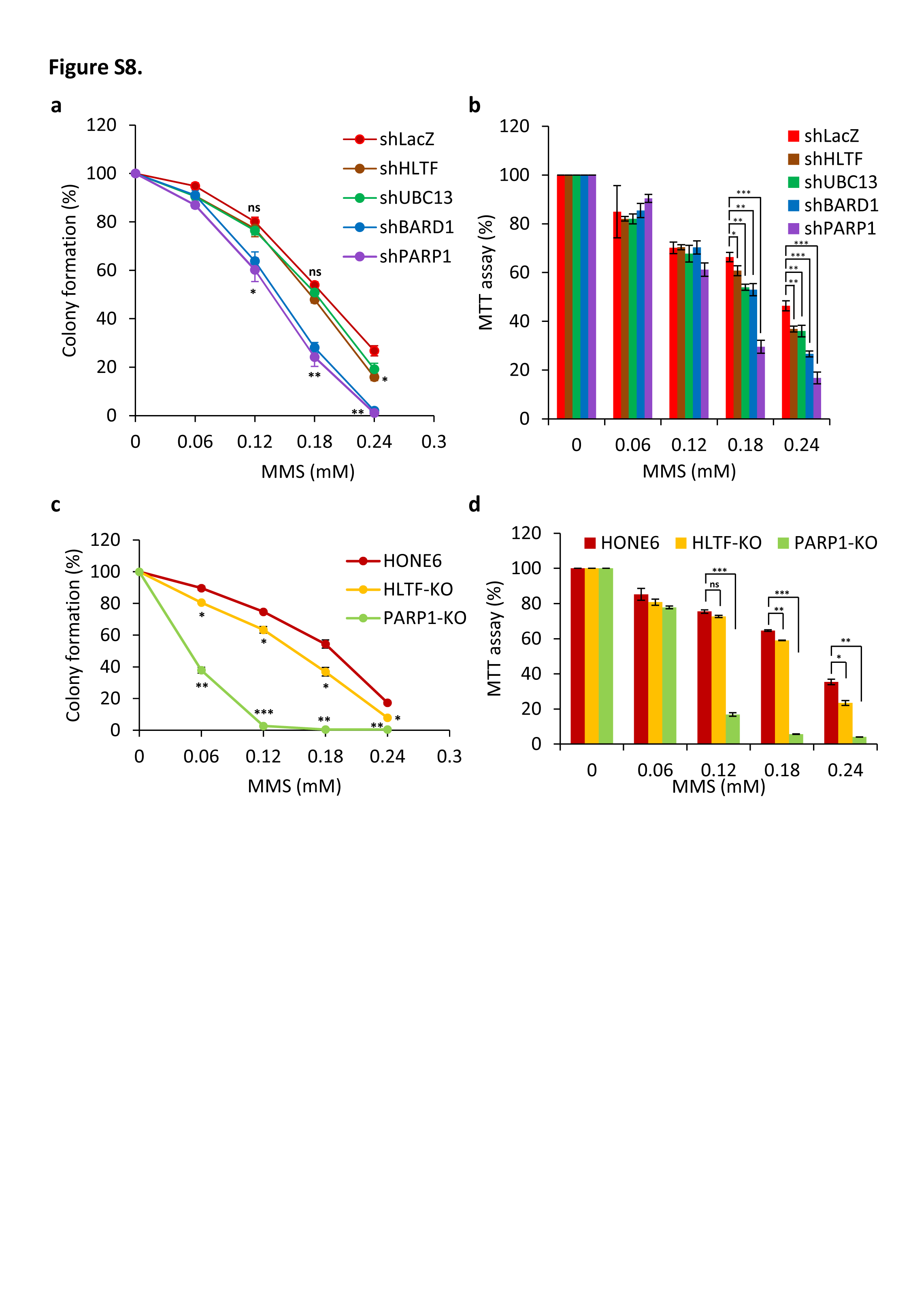

Supplement: Supplementary file 10 — supplementary Figure S8 [file 41389_2020_289_MOESM10_ESM.tif]

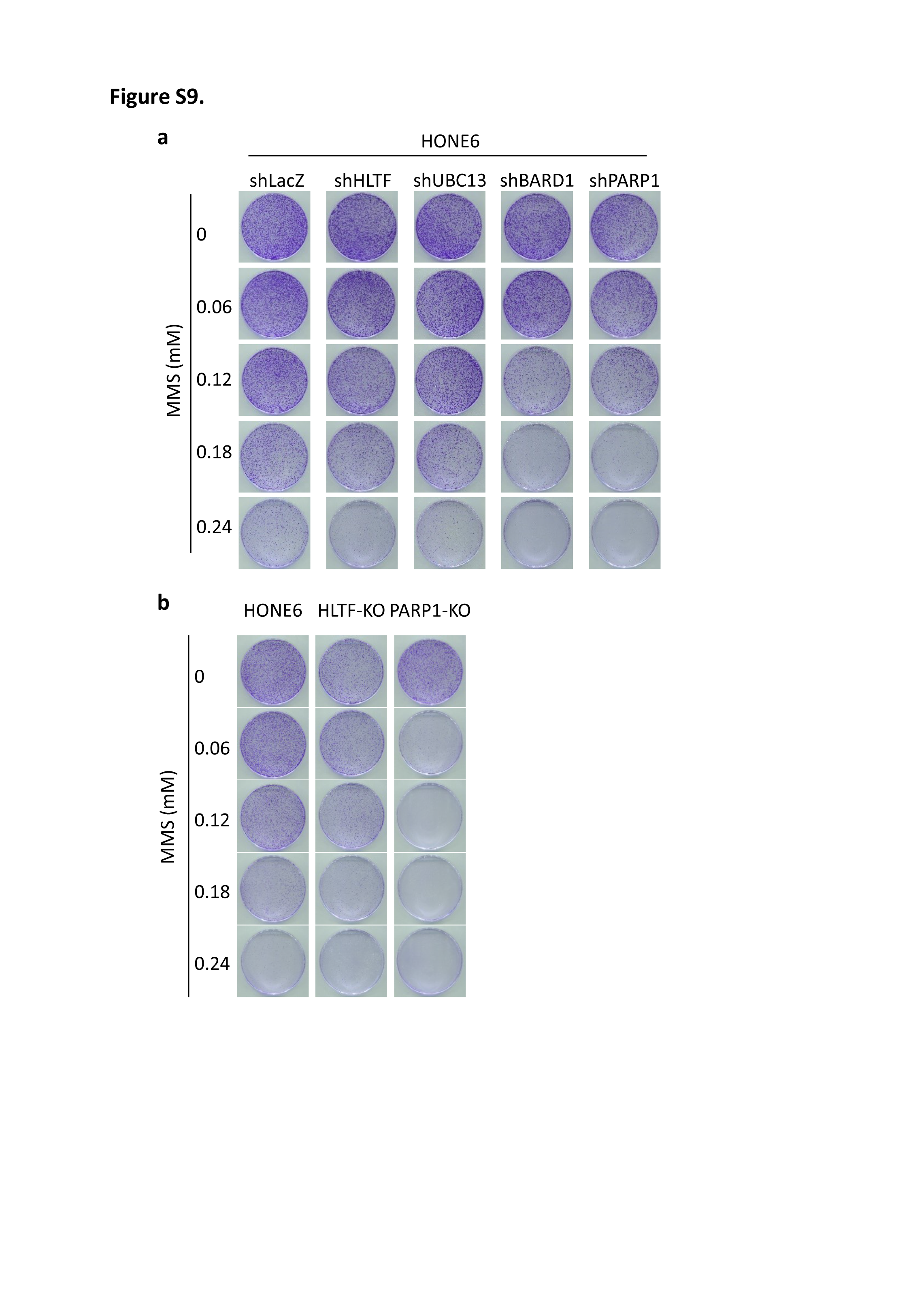

Supplement: Supplementary file 11 — supplementary Figure S9 [file 41389_2020_289_MOESM11_ESM.tif]

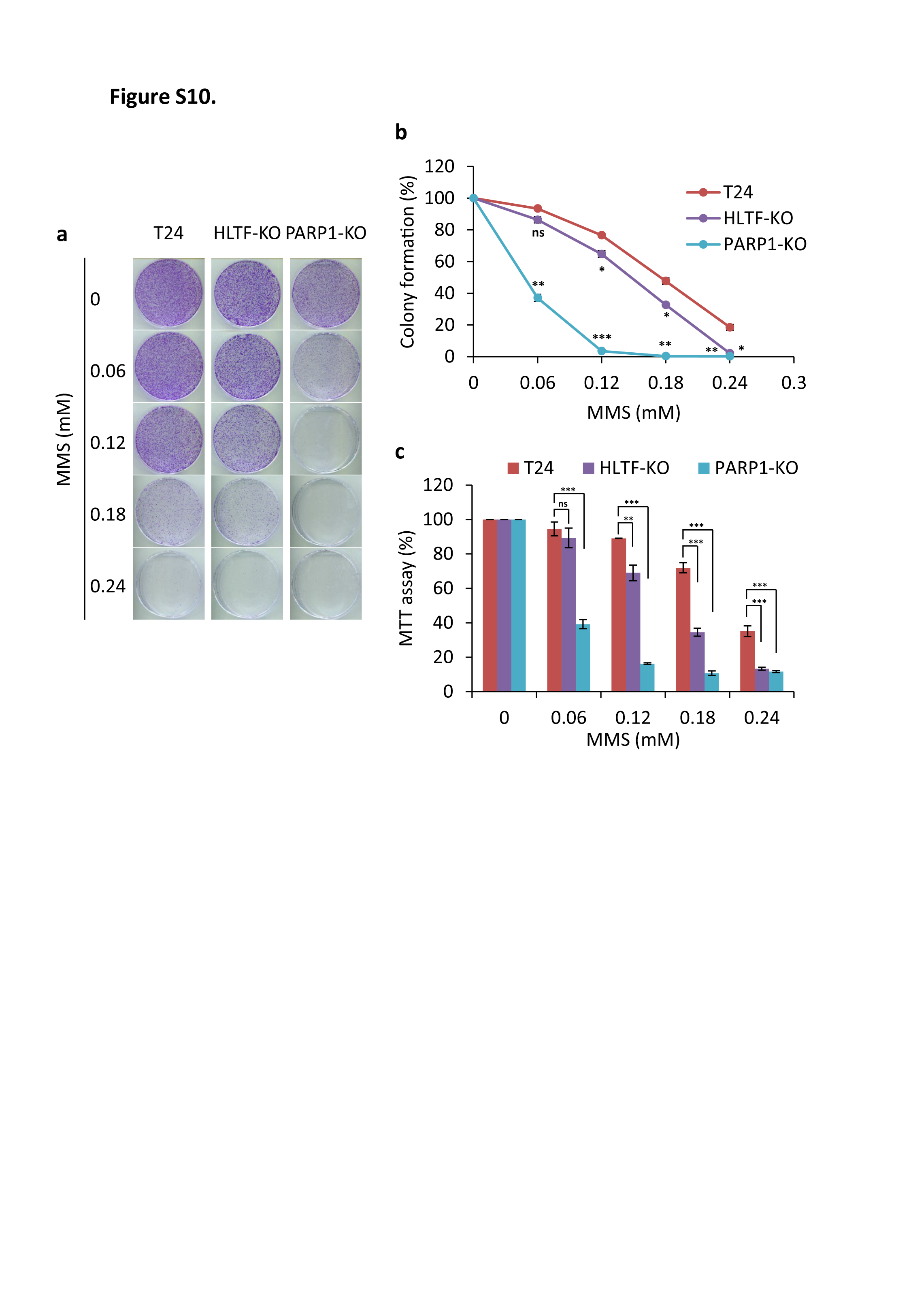

Supplement: Supplementary file 12 — supplementary Figure S10 [file 41389_2020_289_MOESM12_ESM.tif]

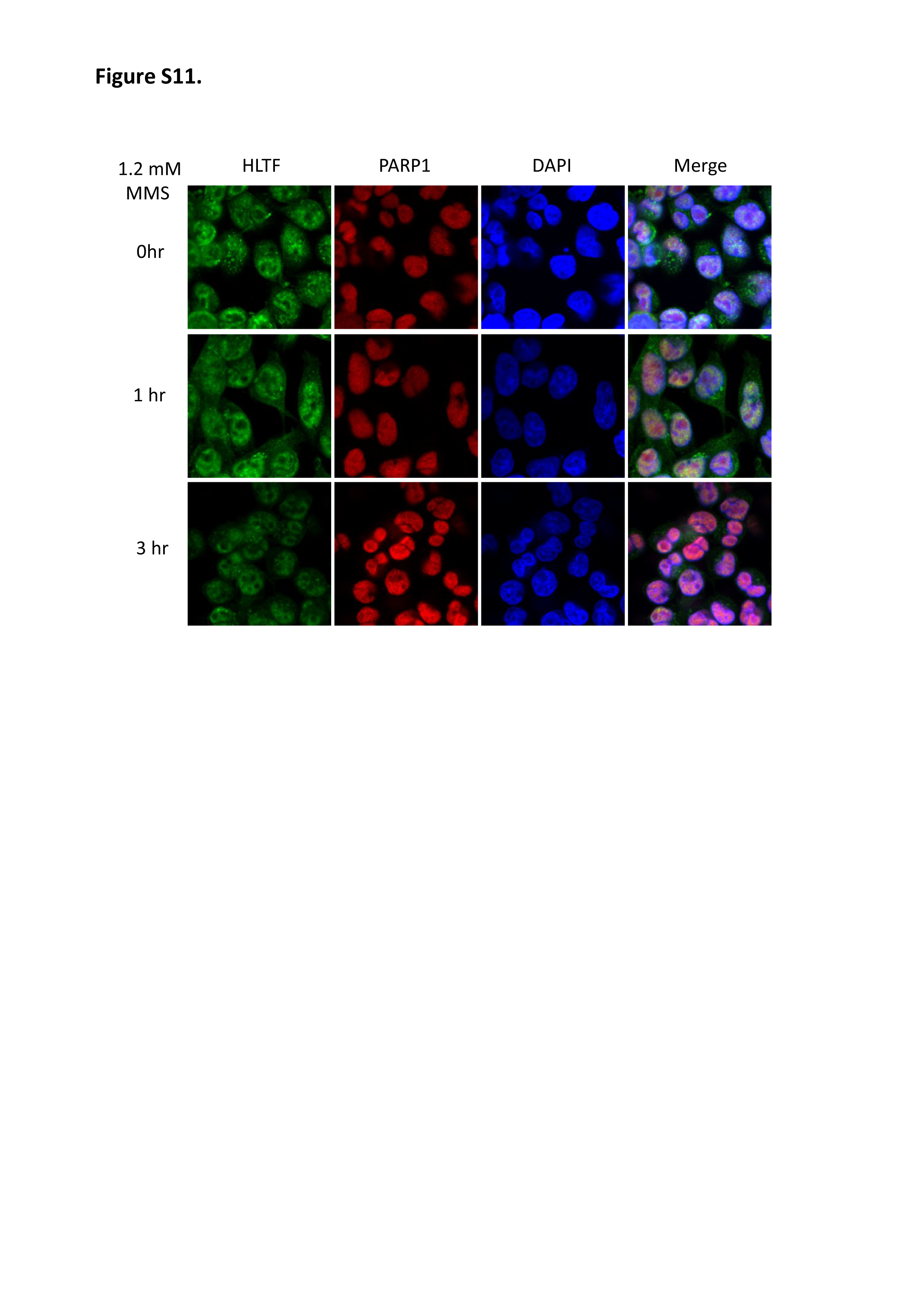

Supplement: Supplementary file 13 — supplementary Figure S11 [file 41389_2020_289_MOESM13_ESM.tif]

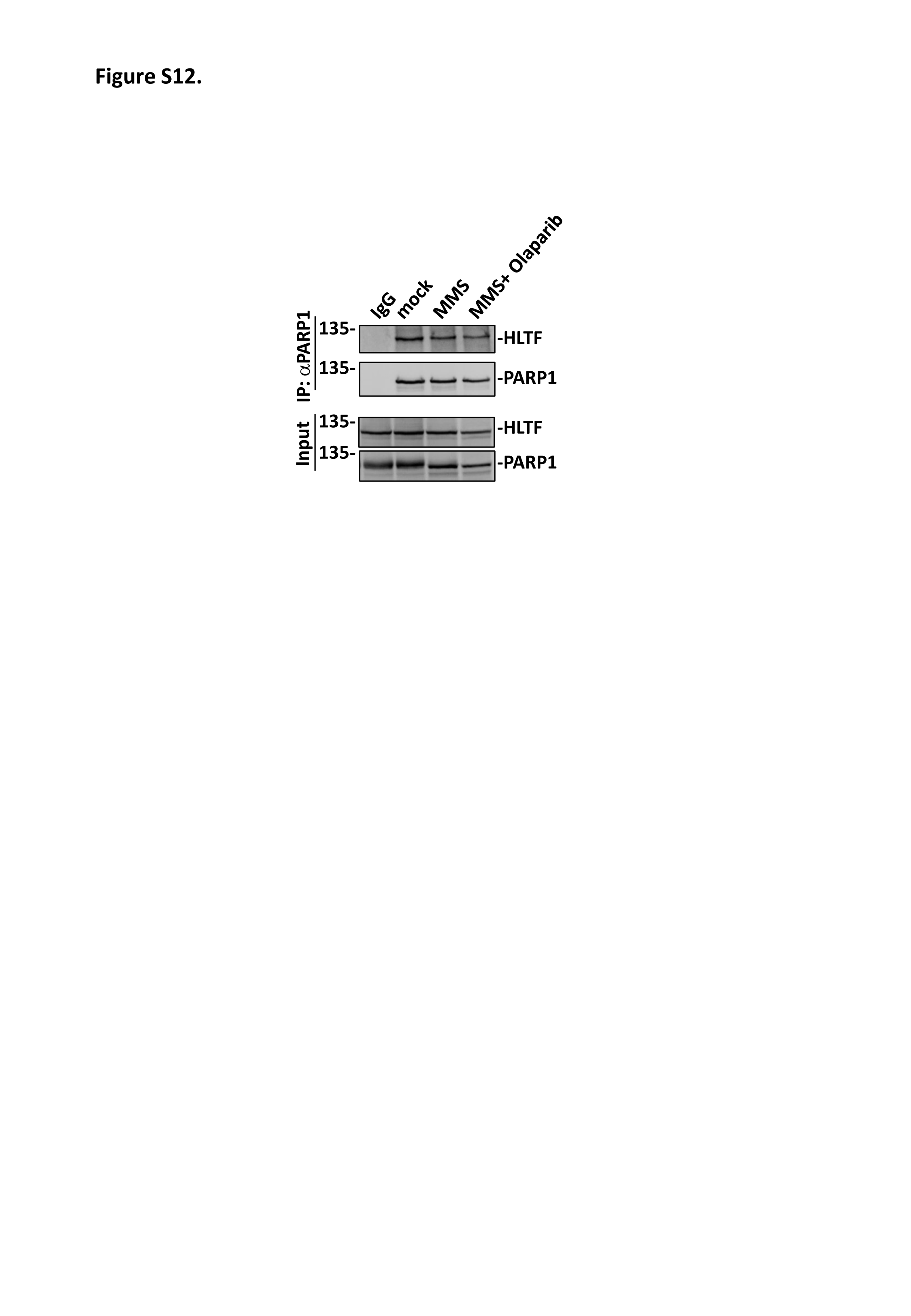

Supplement: Supplementary file 14 — supplementary Figure S12 [file 41389_2020_289_MOESM14_ESM.tif]

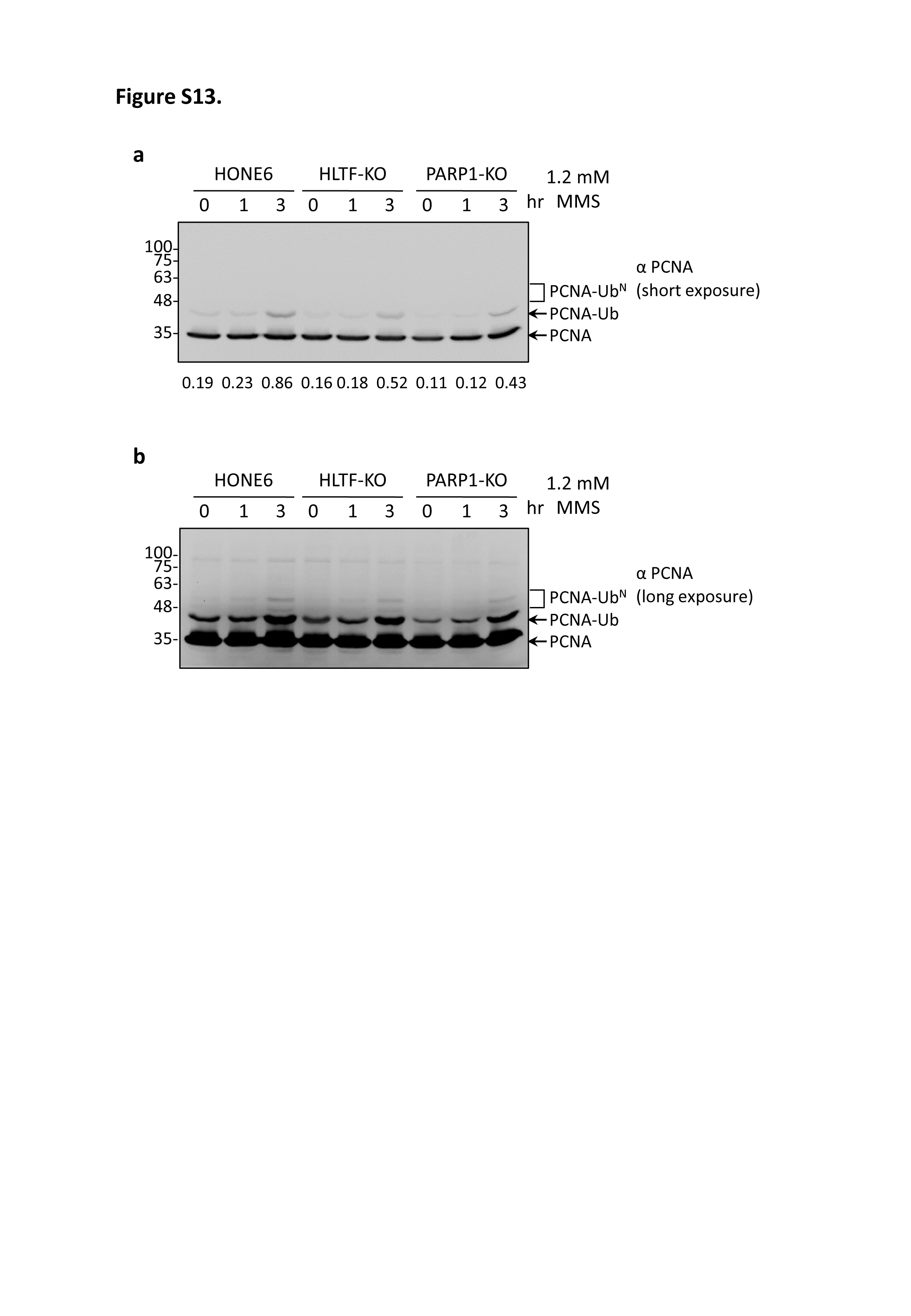

Supplement: Supplementary file 15 — supplementary Figure S13 [file 41389_2020_289_MOESM15_ESM.tif]

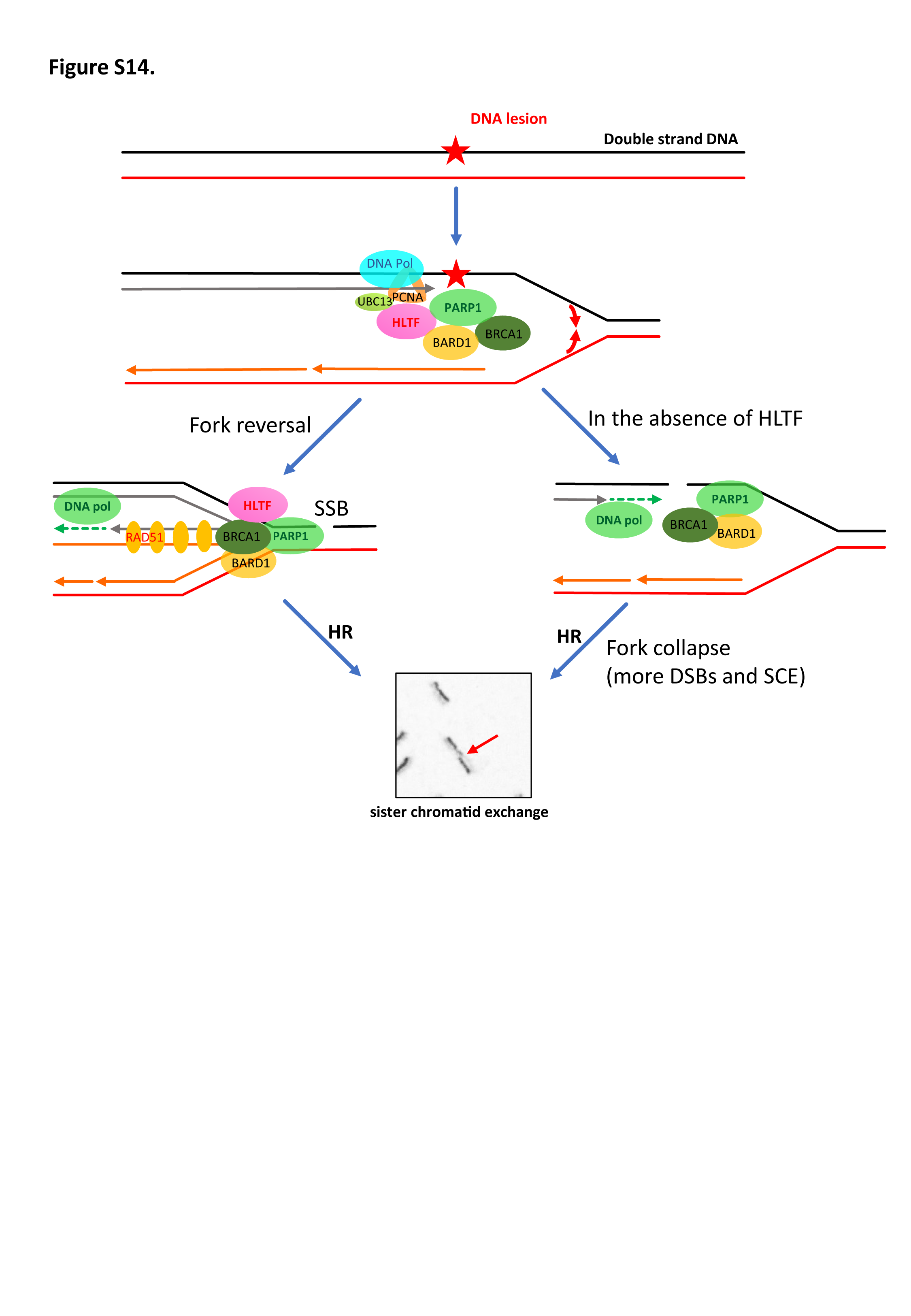

Supplement: Supplementary file 16 — Supplementary Figure S14 [file 41389_2020_289_MOESM16_ESM.tif]
