# Supplementary material for: The HLTF–PARP1 interaction in the progression and stability of damaged replication forks caused by methyl methanesulfonate
Source: Oncogenesis. 2020 Dec 7;9(12):104. doi: 10.1038/s41389-020-00289-5 (PMC7719709; doi:10.1038/s41389-020-00289-5)
Supplement: Supplementary file 17 — supplementary Table S1 [file 41389_2020_289_MOESM17_ESM.pdf]

Table S1. The list of proteins identified in mass spectrometry.

ANM5
CASC1
CCAR1
DDX1
DDX5
DHX9
EF2
EIF3A
EIF3B
EIF3C
FLNA
HNRPL
HNRPU
HSP71
IF4B
IMB1
MYH9
NUCL
PARP1
PRKDC
PSMD2
RAD50
RBM10
SPTA2
SPTB2
SRRT
XRCC5
XRCC6
