# Supplementary material for: The HLTF–PARP1 interaction in the progression and stability of damaged replication forks caused by methyl methanesulfonate
Source: Oncogenesis. 2020 Dec 7;9(12):104. doi: 10.1038/s41389-020-00289-5 (PMC7719709; doi:10.1038/s41389-020-00289-5)
Supplement: Supplementary file 18 — supplementary Table S2 [file 41389_2020_289_MOESM18_ESM.pdf]

Table S2. The list of targeting sequences of shRNA used in this study.

Gene Symbol	Target sequence	Clone ID
LacZ	CGC GAT CGT AAT CAC CCG AGT	TRCN0000231722
HLTF	GCA GGT GGA GTT GGT TTG AAT	TRCN0000272618
HLTF #2	TGT GGT TGG ACT ACG CTA TTA	TRCN0000272562
PARP1	TTT GGT AAA GGG ATC TAT TTC	TRCN0000390913
PARP1 #2	GCA GCT TCA TAA CCG AAG ATT	TRCN0000338406
BARD1	GTC TGC GGC CTG TCG ATT ATA	TRCN0000350414
BARD1 #2	TGG TTT AGC CCT CGA AGT AAG	TRCN0000369045
UBC13/UBE2N	AGA CAA GTT GGG AAG AAT ATG	TRCN0000368937
UBC13/UBE2N #2	CCA TAG AAA CAG CTA GAG CAT	TRCN0000007215
BRCA1	GAG TAT GCA AAC AGC TAT AAT	TRCN0000244984
