# Supplementary material for: The HLTF–PARP1 interaction in the progression and stability of damaged replication forks caused by methyl methanesulfonate
Source: Oncogenesis. 2020 Dec 7;9(12):104. doi: 10.1038/s41389-020-00289-5 (PMC7719709; doi:10.1038/s41389-020-00289-5)
Supplement: Supplementary file 19 — supplementary Table S3 [file 41389_2020_289_MOESM19_ESM.pdf]

Table S3. The list of antibodies used in this study.

Antibodies	WB Dilute concentration	PLA Dilute concentration	Catalog number	Source
Rabbit polyclonal anti-HLTF	1:2000	1:100	A300-230A	Bethyl
Mouse monoclonal anti-PARP1	1:500	1:50	556494	BD Biosciences
Mouse monoclonal anti-BARD1	1:50	1:10	ab50984	Abcam
Rabbit polyclonal anti-BARD1	1:2000	1:100	A300-263A	Bethyl
Mouse monoclonal anti-BARD1	1:1000	1:50	SC-74559	Santa cruz
Rabbit polyclonal anti-UBC13	1:1000	1:200	ab25885	Abcam
Mouse monoclonal anti-BRCA1	1:2000	1:200	ab16780	Abcam
Rabbit polyclonal anti-RAD51	-	1:100	ab63801	Abcam
Mouse monoclonal anti-PCNA	-	1:100	CBL407	Millipore
Mouse monoclonal anti-γH2AX	1:5000	-	05-636	Millipore
Rabbit polyclonal anti-H2AX	1:5000	-	07-627	Millipore
Rabbit monoclonal anti-beta-tubulin	1:5000	-	ab205790	Abcam
Mouse monoclonal anti-biotin	-	1:2000	200-002-211	Jackson ImmunoResearch
Rabbit polyclonal anti-biotin	-	1:3000	A150-109A	Bethyl
Mouse monoclonal anti-EB1	-	1:100	610534	BD Biosciences
Mouse monoclonal anti-FLAG	1:15000	-	F3165	Sigma-Aldrich
Rabbit polyclonal anti-GFP	1:2000	-	ab290	Abcam
Mouse monoclonal anti-turboGFP	1:2000	-	TA150041	Origene
Mouse monoclonal anti-alpha tubulin	1:5000	-	GTX628802-01	Genetex
Rabbit polyclonal anti-53BP1	1:1000	1:100	A5757	ABclonal
